# *Euglena gracilis* Suppresses Cold Symptoms in Healthy Individuals: A Double‐Blind, Randomized, Placebo‐Controlled Trial

**DOI:** 10.1002/fsn3.70935

**Published:** 2025-09-18

**Authors:** Ayaka Nakashima, Kengo Suzuki, Masafumi Nagata, Tsuyoshi Takara

**Affiliations:** ^1^ Euglena Co. Ltd. Tokyo Japan; ^2^ Nerima Medical Association Minami‐Machi Clinic Tokyo Japan; ^3^ Medical Corporation Seishinkai Takara Clinic Tokyo Japan

**Keywords:** common cold, *Euglena gracilis*, immune system, microalgae, paramylon, virus

## Abstract

The immune system functions to eliminate pathogens, such as viruses, and regulate physiological responses and pathways. General immunity is affected by various factors, including fatigue, stress, and aging. A weakened immune system reduces the ability of the body to eliminate pathogens, thereby increasing the risk of infections, including the common cold. *Euglena gracilis* is a microalga rich in various nutrients, including β‐1,3‐glucan paramylon, which aids in maintaining and regulating immune function. In the present study, we aimed to investigate the effects of *E. gracilis* administration on cold symptoms in healthy individuals. *Euglena gracilis* was administered orally in capsule form (once daily, in the morning) continuously for 8 weeks to healthy adult Japanese men and women. Evaluation of the effects of *E. gracilis* administration on alleviating cold symptoms revealed significantly fewer cumulative days with cold‐like symptoms over 8 weeks in the *E. gracilis* group than those in the placebo group. Participants in the *E. gracilis* group also demonstrated significantly fewer severe cold‐like symptoms, such as nasal congestion, sore throat, fatigue, and myalgia, than the placebo group in the second half of the trial (weeks 5–8). In conclusion, this study demonstrated that the continuous intake of *E. gracilis* could suppress cold‐like symptoms. Moreover, the consumption of the test food under the conditions used in this study was deemed safe.

## Graphical abstract

In this double‐blind, randomized, placebo‐controlled trial, 8 weeks of *Euglena gracilis* supplementation significantly reduced the cumulative number and severity of cold‐like symptoms in healthy Japanese adults. These effects are likely attributed to the immunomodulatory action of paramylon, a β‐glucan contained in *E. gracilis* .

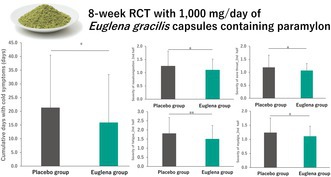

## 1 Introduction

Viruses trigger the symptoms associated with the common cold (common cold syndrome) by infecting the pharyngeal mucosa and replicating within the mucosal tissue. These symptoms, such as runny nose, sneezing, nasal congestion, and sore throat, are commonly observed in the general population, including individuals who are otherwise considered healthy. However, these symptoms can lead to significant economic and psychological consequences, as individuals may be required to miss work or school (Bramley et al. 2002).

*Euglena gracilis* is a unicellular microorganism classified as a microalga. *E. gracilis* , which exhibits both plant and animal properties, contains a myriad of nutrients, including vitamins, minerals, amino acids, and fatty acids. Since the introduction of large‐scale cultivation technology for *E. gracilis* in 2005 for dietary purposes, it has been widely used as a dietary and nutritional supplement (Suzuki 2017). Based on established production data, the nutritional composition of *E. gracilis* per gram includes moisture (≥ 33 mg, by heat drying), protein (≥ 256 mg, by combustion), lipid (≥ 93 mg, by diethyl ether extraction), ash (≥ 31 mg, by direct ashing), and carbohydrate (≥ 433 mg, calculated by difference).

Paramylon, an insoluble β‐glucan, typically accounts for approximately 70%–80% of the carbohydrate content (Shimada et al. 2016; Nakashima et al. 2017; Yasuda et al. 2020; Dai et al. 2022). A notable nutrient and the principal component of *Euglena*, β‐1,3‐glucan paramylon is a storage polysaccharide known for its effects in alleviating atopic dermatitis and providing hepatoprotection (Sugiyama et al. 2009, 2010). In a mouse model, the intake of paramylon powder alleviated the symptoms associated with influenza infections (Nakashima et al. 2017). In healthy individuals, the consumption of *E. gracilis* EOD‐1, which is rich in paramylon, for 4 weeks markedly increased the concentration and secretion rate of salivary secretory IgA (sIgA) (Ishibashi et al. 2019). These data indicate that paramylon plays a key role in immune function.

Dectin‐1 is the main receptor for β‐glucans and is expressed on intestinal immune cells, including dendritic cells (DCs) and macrophages. Upon recognition by Dectin‐1, paramylon is internalized by cells, activating the tyrosine kinase SYK and the transcription factor NF‐κB, thereby promoting cytokine secretion (Nakashima et al. 2018; Ujita et al. 2009). Paramylon may also act directly on DCs, which are antigen‐presenting cells, in the intestine (Yasuda et al. 2020). These findings suggest that paramylon exerts its immunological effects through Dectin‐1.

The autonomic nervous system and immune functions are closely interconnected. Specifically, immune function is enhanced under parasympathetic dominance, aiding in defense against infections (Piccirillo et al. 2004). However, factors such as fatigue, stress, and lack of sleep can disrupt this balance, diminishing immune function. Furthermore, consumption of 1000 mg of *E. gracilis* has been shown to regulate autonomic balance, reduce irritability and tension, and enhance sleep quality (Nakashima et al. 2020), indirectly supporting immune function maintenance. Several studies have indicated the potential of *E. gracilis* containing paramylon to alleviate cold symptoms (Evans et al. 2019; Kawano et al. 2023); however, large‐scale investigations involving Japanese populations have yet to be conducted.

We hypothesized that *Euglena* intake suppresses cold symptoms by modulating the immune balance. Therefore, in the present study, we aimed to investigate the effects of *E. gracilis* consumption on cold symptoms in a population of healthy Japanese men and women.

## 2 Materials and Methods

### 2.1 Materials

*Euglena gracilis* powder was obtained from Euglena Co. Ltd. (Tokyo, Japan). The *Euglena* used in this study was derived from the *Euglena gracilis* Z strain. The placebo capsules contained the same combination of agents without *E. gracilis* powder.

### 2.2 Intake Study

#### 2.2.1 Target Demographics

The study involved 213 healthy Japanese adults, including 68 men and 145 women, aged between 21 and 82 years. After the participants received a sufficient explanation of the objective and content of the study, both verbally and in writing, they provided written informed consent to participate. The present study was conducted in accordance with the tenets of the Declaration of Helsinki and the Ethical Guidelines for Medical and Health Research Involving Human Subjects. The rights of the participants were upheld throughout the study. The study was approved by the Institutional Review Board of Takara Clinic (approval no. 2112‐00395: 2112‐00395‐0022‐1C‐TC, approval date: 12/24/2021). This trial is registered at UMIN ID: 000046464.

#### 2.2.2 Trial Methods

This study was designed as a double‐blind, randomized, placebo‐controlled trial. The participants were selected through screening based on the severity of their typical cold symptoms and fatigue levels.

An independent staff member not involved in the trial randomly assigned the participants to two groups using a computer‐generated random number table. The assignment was conducted considering age, sex, typical cold symptoms, specific IgE levels for cedar and cypress pollen, and fatigue levels to minimize variability between the groups. Blinding was applied to all individuals involved in the trial, including participants, intervention implementers, and evaluators. The allocation table remained blinded until the analysis population was finalized. Access to the allocation table was restricted to the principal investigator, and no other organizations or individuals were able to view it until the allocation manager provided it after the completion of the trial.

Participants were advised against consuming alcohol and exercising excessively from the day before testing until the end of the testing. Additionally, they were instructed to abstain from food and drinks, including the test food, for 6 h before blood collection. However, water intake was permitted. Baseline measurements were recorded during clinic visits prior to intake testing (0 week), which included visual analog scale (VAS) administration, blood collection, and physical measurements such as height, body weight, and body mass index. Cold symptoms during the 5 days before pre‐intake testing were recorded with dietary recall. Following these baseline measurements (0 week), the participants consumed capsules containing 1000 mg of *E. gracilis* powder or placebo powder (starch) once daily for 8 weeks and recorded their cold symptoms in a diary. Participants typically took the capsules in the morning; if they forgot, they took them later in the day. After 8 weeks of intake, the participants returned to the clinic for the same measurements conducted at baseline. Participants were enrolled in the study between January 31 and May 1, 2022, and completed the study between March 28 and June 26, 2022, at a clinic in Tokyo, Japan.

#### 2.2.3 Survey of Cold Symptoms

During the trial period, participants rated general malaise, chills, feverishness, fatigue, sneezing, runny nose, nasal congestion, sore throat, coughing, arthralgia, myalgia, and headache daily in a diary on the following five‐point scale: (1) Normal; (2) Slight; (3) Mild; (4) Moderate; and (5) Severe. The diaries were mailed weekly by post. The final diary was submitted directly to the clinic during final testing. Responses of 3–5 were assessed as the presence of symptoms. “Cold symptoms” were defined as the presence of one or more of the following symptoms: general malaise, chills, fever, fatigue, sneezing, runny nose, nasal congestion, sore throat, coughing, arthralgia, or myalgia. Cumulative days with cold symptoms, the most consecutive days, and symptom severity were also recorded. These figures were calculated from the first day of intake to the end of week 8; the first day to the end of week 4 (Days 1–28) was defined as the first half, whereas the beginning of week 5 to the end of week 8 (Days 29–56) was defined as the second half.

#### 2.2.4 VAS

As immune function is associated with the autonomic nervous system and stress, related parameters that may reflect these factors were further evaluated as supplementary measures. A 100 mm VAS was used to quantify the following on Day 0 (baseline) and Day 56: physical fatigue, mental fatigue, stress, mood, energy, anxiety, tension, depression, relaxedness, irritability, satisfaction with sleep, wake‐up quality, falling asleep, satisfaction with defecation, and refreshment upon defecation. Each VAS question had “0” as an endpoint representing affirmative status and “100” as an endpoint representing negative status. Participants were instructed to mark a point on the line representing their current mood. Responses to the VAS questions were measured as the distance (mm) from the zero endpoint.

### 2.3 Statistical Analysis

The primary outcome was the cumulative days with cold symptoms throughout the trial period; all other outcomes were considered secondary outcomes. Cumulative days, the highest number of consecutive days, and symptom severity are presented as means and standard deviations (SD) and were compared between groups using Welch's *t‐*test. The proportions of individuals with and without symptoms and responses to each question throughout the trial period and during the first and second halves of the trial period were compared between the groups using the chi‐square test, given that these data were categorical. All statistical analyses were performed using two‐sided tests, with the level of statistical significance defined as *p* < 0.05. SPSS Statistics version 23 (SPSS Inc., San Diego, CA, USA) was used for statistical analysis. Given that this trial focused on analyzing the primary outcome, the multiplicity of secondary outcomes was not considered.

The VAS was compared between groups using analysis of covariance (ANCOVA), with Δ defined as the difference between groups in estimated marginal means, the baseline as a covariate, and the group as a factor. Within‐group comparisons were performed using paired *t*‐tests.

## 3 Results

### 3.1 Participants

**FIGURE 1:**
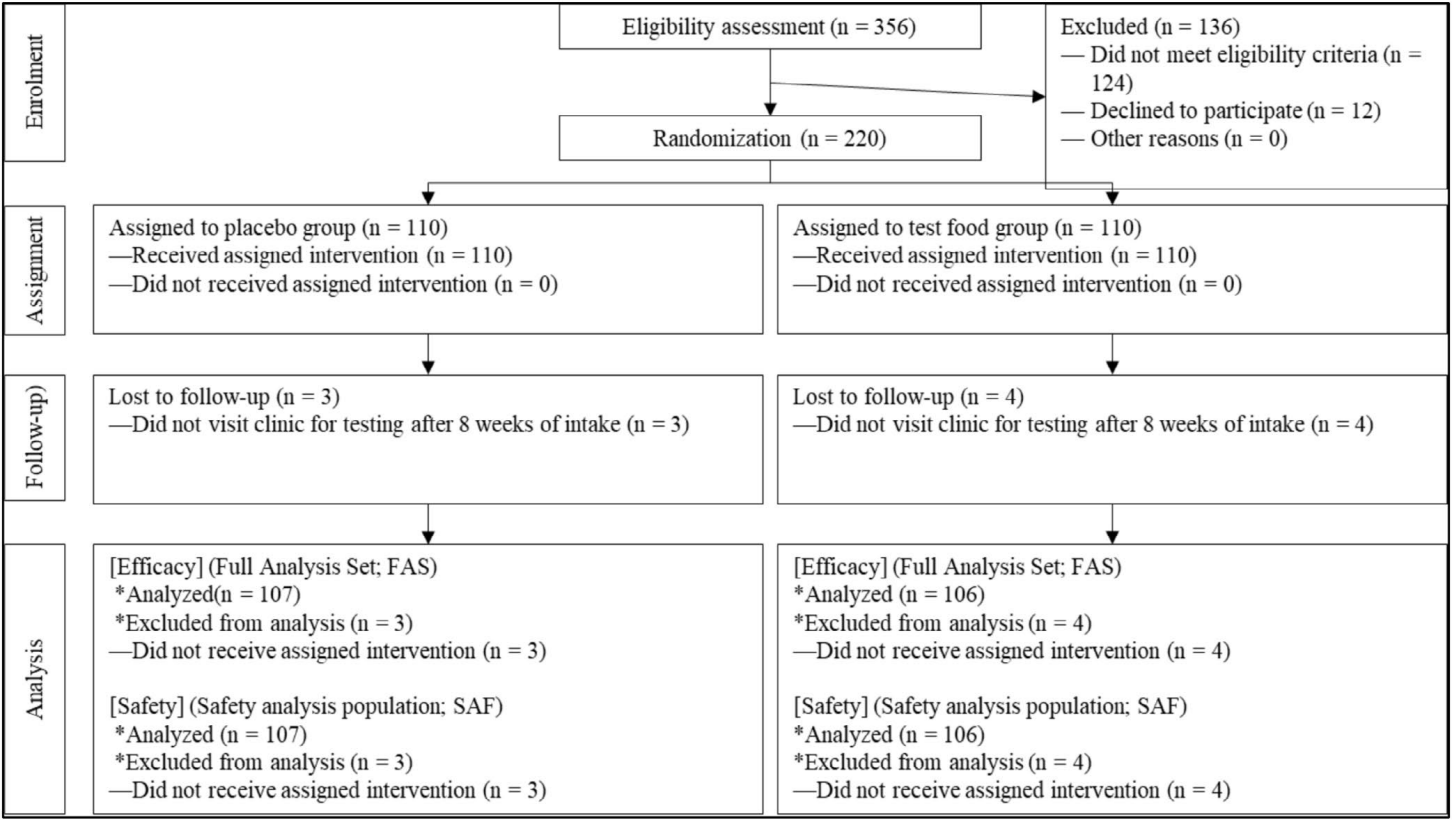
Participant flowchart.

Of the 356 individuals who consented to participate in the trial, 220 met the eligibility criteria and were included in the study (Figure 1) and were assigned to the *Euglena* or placebo groups, with 110 participants in each group. Although all participants received the intervention, seven (three in the placebo group and four in the *Euglena* group) dropped out during the trial. Therefore, data were analyzed for 213 participants (placebo: 107, *Euglena*: 106).

Baseline parameters did not differ significantly between the placebo and *Euglena* groups (Table 1; Tables S1 and S2). Additionally, no differences were observed between the groups in specific IgE class levels for cedar or cypress pollen or cold symptoms 5 days before pre‐intake testing (Table S3).

**TABLE 1:** Baseline data of the participants.

Item	Unit		Placebo group	*Euglena* group
Sex	—	*n*	107	106
		Male	35 (32.7%)	33 (31.1%)
		Female	72 (67.3%)	73 (68.9%)
		*p* value (vs. placebo)^a^	—	0.883
Age	Years	*n*	107	106
		Mean (SD)	46.5 (12.1)	46.3 (11.8)
		Med	48.0	46.0
		Min–Max	21–82	21–80
		*p* value (vs. placebo)^b^	—	0.901
Height	cm	*n*	107	106
		Mean (SD)	163.6 (8.2)	162.3 (8.4)
		Med	163.00	162.00
		Min–Max	147.0–184.0	142.5–181.0
		*p* (vs. placebo)^b^	—	0.247
Body weight	kg	*n*	107	106
		Mean (SD)	60.3 (12.1)	58.4 (12.5)
		Med	59.10	54.50
		Min–Max	40.6–107.4	30.5–90.9
		*p* value (vs. placebo)^b^	—	0.265
BMI	kg/m^2^	*n*	107	106
		Mean (SD)	22.4 (3.4)	22.0 (3.7)
		Med	21.60	21.10
		Min–Max	16.3–35.1	13.0–37.9
		*p* value (vs. placebo)^b^	—	0.471
Systolic blood pressure	mmHg	*n*	107	106
		Mean (SD)	116.6 (13.4)	115.8 (12.3)
		Med	114.00	116.00
		Min–Max	93–148	88–148
		*p* value (vs. placebo)^b^	—	0.653
Diastolic blood pressure	mmHg	*n*	107	106
		Mean (SD)	75.8 (11.0)	75.5 (9.7)
		Med	75.0	74.0
		Min–Max	52–105	54–96
		*p* value (vs. placebo)^b^	—	0.831

Abbreviations: BMI, body mass index; Max, maximum; Med, median; Min, minimum; SD, standard deviation.

^a^ Between‐group comparison with chi‐square test.

^b^ Between‐group comparison with Welch's *t*‐test; *n*, number of participants.

### 3.2 Safety Assessment

No adverse drug reactions were reported during the trial period. Although some participants experienced adverse events, these were determined to be unrelated to the test capsules in all groups (Table S4). The two groups did not exhibit significant differences in the proportion of participants whose urine and peripheral blood test values were within the normal range during screening or baseline testing and subsequently deviated from the normal range after the intervention (Table S5). The investigator verified the safety assessment criteria for each participant, confirming that no medically concerning changes were associated with the test capsules.

### 3.3 Efficacy Assessment

#### 3.3.1 Measurement of Cold Symptoms

The number of cumulative days with cold symptoms throughout the trial (primary outcome) was 21.3 ± 19.2 days in the placebo group compared to 15.9 ± 17.5 days in the *Euglena* group (*p* = 0.032; Table 2). Similarly, as a secondary outcome, the cumulative number of days with cold symptoms in the second half (weeks 5–8) was significantly lower in the *Euglena* group than that in the placebo group (placebo: 11.0 ± 10.4 days, *Euglena*: 7.7 ± 9.3 days, *p* = 0.015). Regarding the cumulative number of days for each cold symptom, the number of days where fatigue was experienced during the second half of the trial was significantly reduced in the *Euglena* group compared to that in the placebo group (6.8 ± 8.8 days vs. 4.5 ± 7.5 days, *p* = 0.042). In terms of symptom severity, the *Euglena* group showed significantly lower severity of feverishness over the entire trial period (1.1 ± 0.3 vs. 1.0 ± 0.2, *p* = 0.040; Table 3), as well as fatigue (1.8 ± 0.9 vs. 1.5 ± 0.7, *p* = 0.007), nasal congestion (1.3 ± 0.6 vs. 1.1 ± 0.4, *p* = 0.029), sore throat (1.2 ± 0.5 vs. 1.1 ± 0.3, *p* = 0.023), and myalgia (1.2 ± 0.5 vs. 1.1 ± 0.4, *p* = 0.036), in the second half than the placebo group.

**TABLE 2:** Cumulative days of cold symptoms overall and for individual symptoms.

Item	Period	Unit	Placebo group						*Euglena* group						Between‐group comparisons				
			*n*	Mean	SD	Med	Min	Max	*n*	Mean	SD	Med	Min	Max	Δ	SE	95% CI−	95% CI+	*p*
Cumulative days with cold symptoms	Overall^a^	Days	107	21.3	19.2	18.0	0.0	56.0	106	15.9	17.5	8.0	0.0	56.0	−5.4	2.5	−10.4	−0.5	0.032*
	1st half	Days	107	10.3	9.9	7.0	0.0	28.0	106	8.2	9.3	4.0	0.0	28.0	−2.1	1.3	−4.7	0.5	0.110
	2nd half	Days	107	11.0	10.4	8.0	0.0	28.0	106	7.7	9.3	3.0	0.0	28.0	−3.3	1.4	−6.0	−0.7	0.015*
Cumulative days with general malaise	Overall	Days	107	6.0	10.3	1.0	0.0	54.0	106	5.6	11.6	0.0	0.0	56.0	−0.4	1.5	−3.4	2.6	0.783
	1st half	Days	107	2.8	4.8	0.0	0.0	26.0	106	3.2	6.4	0.0	0.0	28.0	0.4	0.8	−1.2	1.9	0.630
	2nd half	Days	107	3.2	6.0	0.0	0.0	28.0	106	2.4	5.8	0.0	0.0	28.0	−0.8	0.8	−2.4	0.8	0.332
Cumulative days with chills	Overall	Days	107	1.1	3.6	0.0	0.0	30.0	106	1.0	2.7	0.0	0.0	20.0	−0.2	0.4	−1.0	0.7	0.683
	1st half	Days	107	0.7	2.7	0.0	0.0	26.0	106	0.7	2.4	0.0	0.0	20.0	0.1	0.4	−0.6	0.7	0.879
	2nd half	Days	107	0.5	1.3	0.0	0.0	7.0	106	0.3	0.8	0.0	0.0	6.0	−0.2	0.2	−0.5	0.1	0.130
Cumulative days with feverishness	Overall	Days	107	0.9	2.2	0.0	0.0	16.0	106	0.9	3.0	0.0	0.0	28.0	0.0	0.4	−0.7	0.7	0.997
	1st half	Days	107	0.3	0.9	0.0	0.0	4.0	106	0.5	2.0	0.0	0.0	18.0	0.2	0.2	−0.3	0.6	0.440
	2nd half	Days	107	0.5	1.6	0.0	0.0	12.0	106	0.4	1.3	0.0	0.0	10.0	−0.2	0.2	−0.6	0.2	0.401
Cumulative days with fatigue	Overall	Days	107	12.8	15.9	6.0	0.0	55.0	106	9.8	14.7	3.0	0.0	56.0	−3.0	2.1	−7.1	1.1	0.153
	1st half	Days	107	6.0	7.8	2.0	0.0	27.0	106	5.3	7.7	2.0	0.0	28.0	−0.7	1.1	−2.8	1.4	0.497
	2nd half	Days	107	6.8	8.8	2.0	0.0	28.0	106	4.5	7.5	1.0	0.0	28.0	−2.3	1.1	−4.5	−0.1	0.042*
Cumulative days with sneezing	Overall	Days	107	4.3	9.0	0.0	0.0	43.0	106	3.5	8.8	0.0	0.0	52.0	−0.8	1.2	−3.2	1.6	0.494
	1st half	Days	107	1.9	4.8	0.0	0.0	23.0	106	1.4	4.1	0.0	0.0	25.0	−0.5	0.6	−1.7	0.8	0.461
	2nd half	Days	107	2.4	5.4	0.0	0.0	24.0	106	2.0	5.7	0.0	0.0	27.0	−0.4	0.8	−1.9	1.1	0.614
Cumulative days with a runny nose	Overall	Days	107	6.5	12.5	0.0	0.0	55.0	106	5.0	11.6	0.0	0.0	56.0	−1.5	1.7	−4.7	1.8	0.370
	1st half	Days	107	2.9	6.4	0.0	0.0	27.0	106	2.4	6.0	0.0	0.0	28.0	−0.5	0.9	−2.2	1.2	0.564
	2nd half	Days	107	3.6	7.4	0.0	0.0	28.0	106	2.7	6.5	0.0	0.0	28.0	−1.0	1.0	−2.9	0.9	0.300
Cumulative days with nasal congestion	Overall	Days	107	3.0	7.9	0.0	0.0	40.0	106	1.8	6.4	0.0	0.0	49.0	−1.1	1.0	−3.1	0.8	0.259
	1st half	Days	107	1.2	3.4	0.0	0.0	18.0	106	1.0	3.4	0.0	0.0	25.0	−0.2	0.5	−1.1	0.7	0.659
	2nd half	Days	107	1.8	5.1	0.0	0.0	27.0	106	0.9	3.8	0.0	0.0	26.0	−0.9	0.6	−2.1	0.3	0.144
Cumulative days with sore throat	Overall	Days	107	2.3	7.2	0.0	0.0	56.0	106	1.9	6.3	0.0	0.0	48.0	−0.5	0.9	−2.3	1.3	0.607
	1st half	Days	107	1.2	4.1	0.0	0.0	28.0	106	1.2	4.1	0.0	0.0	24.0	0.0	0.6	−1.1	1.1	0.957
	2nd half	Days	107	1.1	3.6	0.0	0.0	28.0	106	0.6	2.6	0.0	0.0	24.0	−0.5	0.4	−1.4	0.4	0.245
Cumulative days with coughing	Overall	Days	107	2.5	8.4	0.0	0.0	56.0	106	1.4	4.4	0.0	0.0	34.0	−1.1	0.9	−2.9	0.7	0.226
	1st half	Days	107	1.2	4.4	0.0	0.0	28.0	106	0.7	2.2	0.0	0.0	13.0	−0.5	0.5	−1.4	0.4	0.299
	2nd half	Days	107	1.3	4.3	0.0	0.0	28.0	106	0.7	2.6	0.0	0.0	21.0	−0.6	0.5	−1.6	0.3	0.202
Cumulative days with arthralgia	Overall	Days	107	1.9	5.0	0.0	0.0	36.0	106	2.0	7.6	0.0	0.0	55.0	0.0	0.9	−1.7	1.8	0.992
	1st half	Days	107	1.0	3.5	0.0	0.0	24.0	106	1.0	4.1	0.0	0.0	28.0	0.0	0.5	−1.0	1.1	0.943
	2nd half	Days	107	0.9	2.4	0.0	0.0	14.0	106	0.9	3.6	0.0	0.0	27.0	0.0	0.4	−0.9	0.8	0.945
Cumulative days with myalgia	Overall	Days	107	2.9	7.4	0.0	0.0	51.0	106	1.8	6.0	0.0	0.0	41.0	−1.1	0.9	−2.9	0.7	0.235
	1st half	Days	107	1.3	3.7	0.0	0.0	23.0	106	1.1	3.3	0.0	0.0	21.0	−0.2	0.5	−1.2	0.7	0.616
	2nd half	Days	107	1.6	4.1	0.0	0.0	28.0	106	0.7	2.9	0.0	0.0	20.0	−0.9	0.5	−1.8	0.1	0.079
Cumulative days with a headache	Overall	Days	107	3.1	5.8	0.0	0.0	37.0	106	2.7	6.8	0.0	0.0	54.0	−0.4	0.9	−2.1	1.3	0.656
	1st half	Days	107	1.5	3.4	0.0	0.0	20.0	106	1.4	3.4	0.0	0.0	26.0	−0.1	0.5	−1.0	0.8	0.862
	2nd half	Days	107	1.6	3.2	0.0	0.0	17.0	106	1.3	3.8	0.0	0.0	28.0	−0.3	0.5	−1.3	0.6	0.527

*Note:* Between‐group comparisons were conducted with Welch's *t*‐test. *p < 0.05; **p < 0.01.

Abbreviations: Δ, difference between groups (*Euglena* group vs. placebo group); 95% CI−, lower bound of 95% confidence interval; 95% CI+, upper bound of 95% confidence interval; Max, maximum; Med, median; Min, minimum; *n*, number of participants; SD, standard deviation; SE, standard error of the difference between groups.

^a^ Primary outcome.

**TABLE 3:** Severity of cold symptoms overall and for individual symptoms.

Item	Period	Unit	Placebo group						*Euglena* group						Between‐group comparisons				
			*n*	Mean	SD	Med	Min	Max	*n*	Mean	SD	Med	Min	Max	Δ	SE	95% CI−	95% CI+	*p*
Severity of general malaise	Overall	—	107	1.4	0.6	1.0	1.0	4.0	106	1.4	0.6	1.0	1.0	3.0	0.0	0.1	−0.2	0.2	0.821
	1st half	—	107	1.4	0.6	1.0	1.0	4.0	106	1.4	0.7	1.0	1.0	4.0	0.0	0.1	−0.2	0.2	0.800
	2nd half	—	107	1.4	0.7	1.0	1.0	4.5	106	1.3	0.6	1.0	1.0	3.0	−0.1	0.1	−0.2	0.1	0.409
Severity of chills	Overall	—	107	1.1	0.4	1.0	1.0	3.0	106	1.1	0.3	1.0	1.0	2.0	−0.1	0.0	−0.1	0.0	0.173
	1st half	—	107	1.2	0.4	1.0	1.0	3.0	106	1.1	0.4	1.0	1.0	3.0	0.0	0.1	−0.1	0.1	0.672
	2nd half	—	107	1.1	0.3	1.0	1.0	2.0	106	1.1	0.2	1.0	1.0	2.0	−0.1	0.0	−0.1	0.0	0.166
Severity of feverishness	Overall	—	107	1.1	0.3	1.0	1.0	2.0	106	1.0	0.2	1.0	1.0	2.5	−0.1	0.0	−0.2	0.0	0.040*
	1st half	—	107	1.1	0.3	1.0	1.0	2.0	106	1.1	0.3	1.0	1.0	3.0	−0.1	0.0	−0.1	0.0	0.189
	2nd half	—	107	1.1	0.3	1.0	1.0	2.0	106	1.0	0.2	1.0	1.0	2.0	−0.1	0.0	−0.1	0.0	0.081
Severity of fatigue	Overall	—	107	1.7	0.9	2.0	1.0	4.0	106	1.5	0.8	1.0	1.0	4.0	−0.2	0.1	−0.4	0.0	0.095
	1st half	—	107	1.7	0.8	1.5	1.0	4.0	106	1.6	0.8	1.0	1.0	4.0	−0.1	0.1	−0.3	0.1	0.303
	2nd half	—	107	1.8	0.9	2.0	1.0	5.0	106	1.5	0.7	1.0	1.0	4.0	−0.3	0.1	−0.5	−0.1	0.007**
Severity of sneezing	Overall	—	107	1.3	0.5	1.0	1.0	3.0	106	1.2	0.5	1.0	1.0	3.0	−0.1	0.1	−0.2	0.1	0.386
	1st half	—	107	1.3	0.6	1.0	1.0	3.5	106	1.2	0.5	1.0	1.0	3.5	0.0	0.1	−0.2	0.1	0.504
	2nd half	—	107	1.3	0.6	1.0	1.0	3.5	106	1.3	0.6	1.0	1.0	3.5	0.0	0.1	−0.2	0.1	0.585
Severity of runny nose	Overall	—	107	1.4	0.7	1.0	1.0	4.0	106	1.3	0.6	1.0	1.0	5.0	−0.1	0.1	−0.3	0.1	0.202
	1st half	—	107	1.4	0.7	1.0	1.0	4.0	106	1.3	0.7	1.0	1.0	4.0	−0.1	0.1	−0.3	0.1	0.444
	2nd half	—	107	1.4	0.7	1.0	1.0	4.0	106	1.3	0.7	1.0	1.0	5.0	−0.1	0.1	−0.3	0.0	0.131
Severity of nasal congestion	Overall	—	107	1.2	0.5	1.0	1.0	3.0	106	1.1	0.4	1.0	1.0	3.0	−0.1	0.1	−0.2	0.0	0.098
	1st half	—	107	1.2	0.5	1.0	1.0	4.0	106	1.2	0.4	1.0	1.0	3.0	−0.1	0.1	−0.2	0.1	0.386
	2nd half	—	107	1.3	0.6	1.0	1.0	3.0	106	1.1	0.4	1.0	1.0	4.0	−0.1	0.1	−0.3	0.0	0.029*
Severity of sore throat	Overall	—	107	1.2	0.4	1.0	1.0	3.0	106	1.1	0.4	1.0	1.0	3.0	−0.1	0.1	−0.2	0.0	0.157
	1st half	—	107	1.2	0.5	1.0	1.0	4.0	106	1.2	0.4	1.0	1.0	3.0	0.0	0.1	−0.2	0.1	0.618
	2nd half	—	107	1.2	0.5	1.0	1.0	4.0	106	1.1	0.3	1.0	1.0	3.0	−0.1	0.1	−0.2	0.0	0.023*
Severity of coughing	Overall	—	107	1.2	0.5	1.0	1.0	4.0	106	1.1	0.3	1.0	1.0	3.0	−0.1	0.1	−0.2	0.0	0.075
	1st half	—	107	1.2	0.5	1.0	1.0	4.0	106	1.1	0.3	1.0	1.0	2.0	−0.1	0.1	−0.2	0.0	0.152
	2nd half	—	107	1.2	0.5	1.0	1.0	4.0	106	1.1	0.3	1.0	1.0	3.0	−0.1	0.1	−0.2	0.0	0.059
Severity of arthralgia	Overall	—	107	1.2	0.4	1.0	1.0	3.0	106	1.1	0.4	1.0	1.0	4.0	−0.1	0.1	−0.2	0.1	0.359
	1st half	—	107	1.2	0.4	1.0	1.0	3.0	106	1.2	0.5	1.0	1.0	4.0	0.0	0.1	−0.2	0.1	0.508
	2nd half	—	107	1.2	0.4	1.0	1.0	2.5	106	1.1	0.4	1.0	1.0	4.0	−0.1	0.1	−0.2	0.0	0.073
Severity of myalgia	Overall	—	107	1.2	0.5	1.0	1.0	4.0	106	1.1	0.4	1.0	1.0	3.0	−0.1	0.1	−0.2	0.0	0.070
	1st half	—	107	1.2	0.5	1.0	1.0	3.0	106	1.1	0.4	1.0	1.0	3.0	−0.1	0.1	−0.2	0.1	0.284
	2nd half	—	107	1.2	0.5	1.0	1.0	4.0	106	1.1	0.4	1.0	1.0	3.0	−0.1	0.1	−0.3	0.0	0.036*
Severity of headache	Overall	—	107	1.2	0.4	1.0	1.0	3.0	106	1.1	0.4	1.0	1.0	3.0	−0.1	0.1	−0.2	0.1	0.362
	1st half	—	107	1.2	0.4	1.0	1.0	3.0	106	1.2	0.4	1.0	1.0	3.0	0.0	0.1	−0.1	0.1	0.696
	2nd half	—	107	1.2	0.5	1.0	1.0	3.0	106	1.1	0.4	1.0	1.0	3.0	−0.1	0.1	−0.2	0.0	0.161

*Note:* *p < 0.05; **p < 0.01.

Abbreviations: Δ, difference between groups (*Euglena* group versus placebo group); 95% CI−, lower bound of 95% confidence interval; 95% CI+, upper bound of 95% confidence interval; Max, maximum; Med, median; Min, minimum; *n*, number of participants; SD, standard deviation SE, standard error of the difference between groups.

The significant difference observed in the severity of many individual symptoms in the second half of the trial period suggests that *E. gracilis* may be effective when administered continuously for at least 4 weeks. However, the most consecutive days did not differ significantly between the groups for any symptom (Table S6).

The proportion of days with cold symptoms was significantly lower in the *Euglena* group than that in the placebo group throughout the study period (38.0% vs. 28.3%, *p* < 0.001) and in the first (36.9% vs. 29.3%, *p* < 0.001) and second (39.2% vs. 27.3%, *p* < 0.001) halves of the trial (Table 4). Additionally, for individual symptoms such as general malaise, chills, fatigue, sneezing, runny nose, nasal congestion, sore throat, coughing, and muscle pain, the *Euglena* group exhibited a significantly lower proportion of symptomatic days than the placebo group. Additionally, a greater number of significant differences were observed in the later phase than that in the earlier one, suggesting the effectiveness of continuous intake for at least 4 weeks.

**TABLE 4:** Proportions of cumulative days with cold and individual symptoms.

Item	Period	Placebo group			*Euglena* group			Between‐group comparisons				
		Total participant days	Applicable days	Percentage of applicable days (%)	Total participant days	Applicable days	Percentage of applicable days (%)	Δ (%)	95% CI−	95% CI+	χ^2^	*p*
Cumulative days with cold symptoms	Overall	5992	2279	38.0	5936	1682	28.3	−9.7	−11.4	−8.0	126.456	0.000***
	1st half	2996	1105	36.9	2968	871	29.3	−7.5	−9.9	−5.1	38.221	0.000***
	2nd half	2996	1174	39.2	2968	811	27.3	−11.9	−14.3	−9.5	94.457	0.000***
Cumulative days with general malaise	Overall	5992	639	10.7	5936	589	9.9	−0.7	−1.8	0.3	1.776	0.185
	1st half	2996	299	10.0	2968	336	11.3	1.3	−0.2	2.9	2.817	0.102
	2nd half	2996	340	11.3	2968	253	8.5	−2.8	−4.3	−1.3	13.281	0.000***
Cumulative days with chills	Overall	5992	122	2.0	5936	102	1.7	−0.3	−0.8	0.2	1.634	0.225
	1st half	2996	70	2.3	2968	75	2.5	0.2	−0.6	1.0	0.228	0.674
	2nd half	2996	52	1.7	2968	27	0.9	−0.8	−1.4	−0.2	7.782	0.006**
Cumulative days with feverishness	Overall	5992	93	1.6	5936	92	1.5	0.0	−0.4	0.4	0.000	1.000
	1st half	2996	37	1.2	2968	54	1.8	0.6	0.0	1.2	3.389	0.072
	2nd half	2996	56	1.9	2968	38	1.3	−0.6	−1.2	0.0	3.332	0.077
Cumulative days with fatigue	Overall	5992	1368	22.8	5936	1036	17.5	−5.4	−6.8	−3.9	53.587	0.000***
	1st half	2996	645	21.5	2968	562	18.9	−2.6	−4.6	−0.6	6.212	0.013*
	2nd half	2996	723	24.1	2968	474	16.0	−8.2	−10.2	−6.1	61.913	0.000***
Cumulative days with sneezing	Overall	5992	460	7.7	5936	367	6.2	−1.5	−2.4	−0.6	10.319	0.001**
	1st half	2996	203	6.8	2968	153	5.2	−1.6	−2.8	−0.4	6.977	0.009**
	2nd half	2996	257	8.6	2968	214	7.2	−1.4	−2.7	0.0	3.835	0.055
Cumulative days with a runny nose	Overall	5992	699	11.7	5936	535	9.0	−2.7	−3.7	−1.6	22.624	0.000***
	1st half	2996	309	10.3	2968	254	8.6	−1.8	−3.2	−0.3	5.377	0.021*
	2nd half	2996	390	13.0	2968	281	9.5	−3.5	−5.2	−1.9	18.815	0.000***
Cumulative days with nasal congestion	Overall	5992	317	5.3	5936	196	3.3	−2.0	−2.7	−1.3	28.648	0.000***
	1st half	2996	125	4.2	2968	102	3.4	−0.7	−1.7	0.2	2.203	0.155
	2nd half	2996	192	6.4	2968	94	3.2	−3.2	−4.3	−2.2	34.313	0.000***
Cumulative days with sore throat	Overall	5992	250	4.2	5936	197	3.3	−0.9	−1.5	−0.2	6.022	0.016*
	1st half	2996	130	4.3	2968	132	4.4	0.1	−0.9	1.1	0.042	0.850
	2nd half	2996	120	4.0	2968	65	2.2	−1.8	−2.7	−0.9	16.346	0.000***
Cumulative days with coughing	Overall	5992	268	4.5	5936	147	2.5	−2.0	−2.7	−1.3	35.384	0.000***
	1st half	2996	131	4.4	2968	77	2.6	−1.8	−2.7	−0.8	14.006	0.000***
	2nd half	2996	137	4.6	2968	70	2.4	−2.2	−3.1	−1.3	21.819	0.000***
Cumulative days with arthralgia	Overall	5992	208	3.5	5936	207	3.5	0.0	−0.6	0.7	0.002	1.000
	1st half	2996	107	3.6	2968	110	3.7	0.1	−0.8	1.1	0.077	0.783
	2nd half	2996	101	3.4	2968	97	3.3	−0.1	−1.0	0.8	0.049	0.829
Cumulative days with myalgia	Overall	5992	313	5.2	5936	193	3.3	−2.0	−2.7	−1.2	28.555	0.000***
	1st half	2996	141	4.7	2968	114	3.8	−0.9	−1.9	0.2	2.728	0.109
	2nd half	2996	172	5.7	2968	79	2.7	−3.1	−4.1	−2.1	35.067	0.000***
Cumulative days with a headache	Overall	5992	327	5.5	5936	283	4.8	−0.7	−1.5	0.1	2.924	0.088
	1st half	2996	156	5.2	2968	146	4.9	−0.3	−1.4	0.8	0.257	0.637
	2nd half	2996	171	5.7	2968	137	4.6	−1.1	−2.2	0.0	3.628	0.061

*Note:* Between‐group comparisons were conducted using the chi‐square test. *p < 0.05, **p < 0.01, ***p < 0.001.

Abbreviations: Δ, difference in proportion of applicable days (*Euglena* group versus placebo group); 95% CI−, lower bound of 95% confidence interval; 95% CI+, upper bound of 95% confidence interval.

Regarding the severity of individual symptoms, the proportion of participants who responded “(1) Normal” to general malaise, chills, feverishness, fatigue, sneezing, runny nose, nasal congestion, sore throat, coughing, arthralgia, myalgia, and headache was significantly higher in the *Euglena* group than that in the placebo group (Table 5; Table S7). Additionally, for general malaise, fatigue, sneezing, nasal congestion, and sore throat, the proportion of participants who responded “(5) Severe” was significantly lower in the *Euglena* group than that in the placebo group.

**TABLE 5:** Proportions of responses regarding symptom severity.

Item	Period	Placebo group			*Euglena* group			Between‐group comparisons				
		Total participant days	Applicable days	Percentage of applicable days (%)	Total participant days	Applicable days	Percentage of applicable days (%)	Δ (%)	95% CI−	95% CI+	χ^2^	*p*
Percentage of severity 1 general malaise	Overall	5992	3943	65.8	5936	4005	67.5	1.7	0.0	3.4	3.719	0.055
	1st half	2996	1953	65.2	2968	1956	65.9	0.7	−1.7	3.1	0.338	0.567
	2nd half	2996	1990	66.4	2968	2049	69.0	2.6	0.2	5.0	4.662	0.033*
Percentage of severity 5 general malaise	Overall	5992	43	0.7	5936	18	0.3	−0.4	−0.7	−0.2	10.064	0.002**
	1st half	2996	10	0.3	2968	7	0.2	−0.1	−0.4	0.2	0.503	0.629
	2nd half	2996	33	1.1	2968	11	0.4	−0.7	−1.2	−0.3	10.875	0.001**
Percentage of severity 1 chills	Overall	5992	5120	85.4	5936	5292	89.2	3.7	2.5	4.9	36.869	0.000***
	1st half	2996	2523	84.2	2968	2573	86.7	2.5	0.7	4.3	7.369	0.007**
	2nd half	2996	2597	86.7	2968	2719	91.6	4.9	3.3	6.5	37.391	0.000***
Percentage of severity 5 chills	Overall	5992	0	0.0	5936	2	0.0	0.0	0.0	0.1	2.019	0.248
	1st half	2996	0	0.0	2968	2	0.1	0.1	0.0	0.2	2.020	0.248
	2nd half	2996	0	0.0	2968	0	0.0	0.0	N.A.	N.A.	N.A.	N.A.
Percentage of severity 1 feverishness	Overall	5992	5194	86.7	5936	5508	92.8	6.1	5.0	7.2	120.617	0.000***
	1st half	2996	2587	86.3	2968	2734	92.1	5.8	4.2	7.3	51.559	0.000***
	2nd half	2996	2607	87.0	2968	2774	93.5	6.4	4.9	8.0	70.276	0.000***
Percentage of severity 5 feverishness	Overall	5992	2	0.0	5936	9	0.2	0.1	0.0	0.2	4.525	0.038*
	1st half	2996	0	0.0	2968	6	0.2	0.2	0.0	0.4	6.063	0.015*
	2nd half	2996	2	0.1	2968	3	0.1	0.0	−0.1	0.2	0.210	0.686
Percentage of severity 1 fatigue	Overall	5992	2775	46.3	5936	3401	57.3	11.0	9.2	12.8	144.055	0.000***
	1st half	2996	1408	47.0	2968	1621	54.6	7.6	5.1	10.2	34.637	0.000***
	2nd half	2996	1367	45.6	2968	1780	60.0	14.3	11.8	16.9	123.110	0.000***
Percentage of severity 5 fatigue	Overall	5992	80	1.3	5936	40	0.7	−0.7	−1.0	−0.3	13.092	0.000***
	1st half	2996	26	0.9	2968	27	0.9	0.0	−0.4	0.5	0.030	0.891
	2nd half	2996	54	1.8	2968	13	0.4	−1.4	−1.9	−0.8	24.987	0.000***
Percentage of severity 1 sneezing	Overall	5992	4475	74.7	5936	4713	79.4	4.7	3.2	6.2	37.449	0.000***
	1st half	2996	2264	75.6	2968	2379	80.2	4.6	2.5	6.7	18.197	0.000***
	2nd half	2996	2211	73.8	2968	2334	78.6	4.8	2.7	7.0	19.266	0.000***
Percentage of severity 5 sneezing	Overall	5992	28	0.5	5936	16	0.3	−0.2	−0.4	0.0	3.173	0.096
	1st half	2996	10	0.3	2968	15	0.5	0.2	−0.2	0.5	1.052	0.324
	2nd half	2996	18	0.6	2968	1	0.0	−0.6	−0.9	−0.3	15.100	0.000***
Percentage of severity 1 runny nose	Overall	5992	4148	69.2	5936	4472	75.3	6.1	4.5	7.7	55.568	0.000***
	1st half	2996	2078	69.4	2968	2254	75.9	6.6	4.3	8.8	32.520	0.000***
	2nd half	2996	2070	69.1	2968	2218	74.7	5.6	3.4	7.9	23.459	0.000***
Percentage of severity 5 runny nose	Overall	5992	28	0.5	5936	52	0.9	0.4	0.1	0.7	7.477	0.007**
	1st half	2996	10	0.3	2968	26	0.9	0.5	0.1	0.9	7.306	0.007**
	2nd half	2996	18	0.6	2968	26	0.9	0.3	−0.2	0.7	1.542	0.229
Percentage of severity 1 nasal congestion	Overall	5992	4813	80.3	5936	5159	86.9	6.6	5.3	7.9	94.364	0.000***
	1st half	2996	2444	81.6	2968	2552	86.0	4.4	2.5	6.3	21.311	0.000***
	2nd half	2996	2369	79.1	2968	2607	87.8	8.8	6.9	10.7	82.869	0.000***
Percentage of severity 5 nasal congestion	Overall	5992	15	0.3	5936	0	0.0	−0.3	−0.4	−0.1	14.879	0.000***
	1st half	2996	9	0.3	2968	0	0.0	−0.3	−0.5	−0.1	8.929	0.004**
	2nd half	2996	6	0.2	2968	0	0.0	−0.2	−0.4	0.0	5.950	0.031*
Percentage of severity 1 sore throat	Overall	5992	4892	81.6	5936	5360	90.3	8.7	7.4	9.9	184.933	0.000***
	1st half	2996	2439	81.4	2968	2623	88.4	7.0	5.1	8.8	56.385	0.000***
	2nd half	2996	2453	81.9	2968	2737	92.2	10.3	8.6	12.0	141.180	0.000***
Percentage of severity 5 sore throat	Overall	5992	12	0.2	5936	11	0.2	0.0	−0.2	0.1	0.035	1.000
	1st half	2996	6	0.2	2968	11	0.4	0.2	−0.1	0.4	1.522	0.235
	2nd half	2996	6	0.2	2968	0	0.0	−0.2	−0.4	0.0	5.950	0.031*
Percentage of severity 1 coughing	Overall	5992	4957	82.7	5936	5272	88.8	6.1	4.8	7.3	90.452	0.000***
	1st half	2996	2476	82.6	2968	2618	88.2	5.6	3.8	7.4	37.046	0.000***
	2nd half	2996	2481	82.8	2968	2654	89.4	6.6	4.9	8.4	54.433	0.000***
Percentage of severity 5 coughing	Overall	5992	4	0.1	5936	6	0.1	0.0	−0.1	0.1	0.419	0.547
	1st half	2996	2	0.1	2968	6	0.2	0.1	−0.1	0.3	2.041	0.177
	2nd half	2996	2	0.1	2968	0	0.0	−0.1	−0.2	0.0	1.982	0.500
Percentage of severity 1 arthralgia	Overall	5992	4813	80.3	5936	5205	87.7	7.4	6.0	8.7	120.159	0.000***
	1st half	2996	2447	81.7	2968	2573	86.7	5.0	3.2	6.9	28.155	0.000***
	2nd half	2996	2366	79.0	2968	2632	88.7	9.7	7.8	11.6	103.506	0.000***
Percentage of severity 5 arthralgia	Overall	5992	7	0.1	5936	3	0.1	−0.1	−0.2	0.0	1.564	0.344
	1st half	2996	1	0.0	2968	0	0.0	0.0	−0.1	0.0	0.991	1.000
	2nd half	2996	6	0.2	2968	3	0.1	−0.1	−0.3	0.1	0.974	0.508
Percentage of severity 1 myalgia	Overall	5992	4731	79.0	5936	5166	87.0	8.1	6.7	9.4	137.560	0.000***
	1st half	2996	2376	79.3	2968	2542	85.6	6.3	4.4	8.3	41.453	0.000***
	2nd half	2996	2355	78.6	2968	2624	88.4	9.8	7.9	11.7	103.956	0.000***
Percentage of severity 5 myalgia	Overall	5992	21	0.4	5936	10	0.2	−0.2	−0.4	0.0	3.811	0.070
	1st half	2996	8	0.3	2968	5	0.2	−0.1	−0.3	0.1	0.666	0.581
	2nd half	2996	13	0.4	2968	5	0.2	−0.3	−0.5	0.0	3.491	0.096
Percentage of severity 1 headache	Overall	5992	4725	78.9	5936	5062	85.3	6.4	5.0	7.8	83.482	0.000***
	1st half	2996	2380	79.4	2968	2489	83.9	4.4	2.5	6.4	19.450	0.000***
	2nd half	2996	2345	78.3	2968	2573	86.7	8.4	6.5	10.4	73.094	0.000***
Percentage of severity 5 headache	Overall	5992	15	0.3	5936	16	0.3	0.0	−0.2	0.2	0.042	0.859
	1st half	2996	6	0.2	2968	3	0.1	−0.1	−0.3	0.1	0.974	0.508
	2nd half	2996	9	0.3	2968	13	0.4	0.1	−0.2	0.4	0.768	0.402

*Note:* Between‐group comparisons were conducted using the chi‐square test. (1) Normal; (2) Slight; (3) Mild; (4) Moderate; (5) Severe. *p < 0.05, **p < 0.01, ***p < 0.001.

Abbreviations: Δ, difference in proportion of applicable days (*Euglena* group versus placebo group); 95% CI−, lower bound of 95% confidence interval; 95% CI+, upper bound of 95% confidence interval.

#### 3.3.2 VAS Analysis

No significant changes were observed in VAS scores (Table S8). However, in the within‐group comparison of the placebo group, the values for wake‐up quality and satisfaction with defecation improved after 8 weeks of intake compared to baseline (Table S9). Comparisons within the *Euglena* group demonstrated that the values for tension and relaxation were significantly lower after 8 weeks of intake than those at baseline (Table S10), indicating improvements. Comparisons within the *Euglena* group also demonstrated that satisfaction with defecation and the feeling of refreshment upon defecation significantly improved after 8 weeks of intake compared to baseline (Table S10).

## 4 Discussion

The present study evaluated the effect of 8 weeks of continuous *E. gracilis* capsule intake on immune function in healthy adult Japanese men and women. Our findings demonstrated that the mean cumulative days with cold symptoms throughout the trial period (primary outcome) were significantly lower in the *Euglena* group than that in the placebo group. Although it is a secondary endpoint, the number of cumulative days with fatigue in the second half of the trial was also significantly lower in the *Euglena* group than that in the placebo group. Moreover, the *Euglena* group demonstrated significantly fewer cumulative days with general malaise, chills, fatigue, sneezing, runny nose, nasal congestion, sore throat, coughing, and myalgia than the placebo group. In terms of symptom severity, feverishness (overall), fatigue (second half), nasal congestion (second half), sore throat (second half), and myalgia (second half) were significantly milder in the *Euglena* group than in the placebo group. The proportion of responses ranked as severity 1 (“Normal” on the cold symptom survey) for general malaise, chills, feverishness, fatigue, sneezing, runny nose, nasal congestion, sore throat, coughing, arthralgia, myalgia, and headache was significantly higher in the *Euglena* group than in the placebo group. These findings suggest that the intake of *E. gracilis* can effectively mitigate cold symptoms.

Immune functions include eliminating pathogens and regulating physiological mechanisms (Kaufmann 2019). However, immune function can be diminished by various factors such as fatigue (Papacosta and Nassis 2011; Tirelli et al. 2013), stress (Seiler et al. 2020), and aging (Brauning et al. 2022), highlighting the importance of maintaining immunity. In the present study, some of the participants were selected based on relatively high levels of physical fatigue. In general, individuals experiencing chronic fatigue exhibit reduced immune function, primarily due to decreased expression and activity of natural killer (NK) cells (Cabanas et al. 2019; Nguyen et al. 2016, 2017). Weakened immunity prevents the body from effectively eliminating invading pathogens and viruses, thereby increasing the risk of developing and intensifying cold‐like symptoms.

Typically, mild cold symptoms include localized nasal and throat symptoms, whereas severe colds present with systemic symptoms, including fever and myalgia (Eccles 2005, 2009). Colds are triggered primarily by viral infections (Wine and Alper 2012), after which symptoms are elicited via two routes (Eccles 2009). In one route, local symptoms, such as sneezing, runny nose, nasal congestion, and coughing, are induced by bradykinin and prostaglandin synthesis in the nasal epithelium. In contrast, in the other route, systemic symptoms, such as fever, myalgia, and headache, occur when Toll‐like receptor (TLR) stimulation induces the release of cytokines from macrophages, neutrophils, and DCs (Eccles 2009). In this study, “cold symptoms” were defined as the presence of one or more of the following: general malaise, chills, feverishness, fatigue, sneezing, runny nose, nasal congestion, sore throat, coughing, arthralgia, and myalgia. Notably, in the present study, the manifestations of general malaise, chills, fatigue, sneezing, runny nose, nasal congestion, sore throat, arthralgia, and myalgia were suppressed by *Euglena* intake. This finding suggests that the regulation of the immune balance associated with *Euglena* intake affected both pathways of cold‐symptom induction, significantly shortening the period of symptom manifestation.

β‐glucans have been reported to alleviate symptoms of the common cold through the modulation of immune functions (Mah et al. 2020). Therefore, we speculated that the improvement in immune function observed in the present study might involve paramylon, a β‐glucan found in *E. gracilis* . Among the various β‐glucan receptors, including Dectin‐1, TLRs, and complement receptor 3 (CR3), paramylon has been reported to bind, at the very least, to Dectin‐1—a major β‐glucan receptor expressed on epithelial cells, macrophages, and DCs (Brown and Gordon 2001; Cohen‐Kedar et al. 2014; Nakashima et al. 2018). This binding triggers tyrosine kinase‐mediated signaling (Chan et al. 2009) and leads to the release of interleukin‐12 (IL‐12) (Komastu et al. 1998), IL‐10 (Ouyang and O'Garra 2019), IL‐6 (Tanaka et al. 2014), and other cytokines (Chan et al. 2009). These cytokines improve the activity of cells that contribute to immune function, such as NK, Th1, and B cells (Komastu et al. 1998; Ouyang and O'Garra 2019; Tanaka et al. 2014). Moreover, a study in which BALB/c mice were fed *E. gracilis* or paramylon and infected with an influenza virus revealed significantly increased survival rates and low viral titers in the lungs of mice fed *E. gracilis* and paramylon (Nakashima et al. 2017). Additionally, intraperitoneal injection of *E. gracilis* and paramylon stimulates DCs in Peyer's patches (Yasuda et al. 2020). A 12‐week intake of paramylon‐rich *E. gracilis* EOD‐1 in healthy adults aged 50–65 years maintained subjective health status and mitigated cold‐related symptoms, evidenced by decreased incidences of chills, fever, headache, cough, and sore throat. This intake also enhanced naïve T cell activation via increased CD28 expression and improved the antigen‐presenting function of monocytes by maintaining CD80 and CD38 expression, suggesting a strengthened immune defense (Kawano et al. 2023). Another study demonstrated that administering *E. gracilis* powder for 8 weeks in Korean adults (aged 20–70 years) significantly enhanced NK cell activity, demonstrating improved immune function (Park et al. 2023). Moreover, in a population predominantly comprising Western European individuals, with some Eastern European and South Asian representation (21–65 years), who engaged in intense daily endurance exercise and consumed β‐1,3‐glucan‐rich *E. gracilis* for 90 days, the incidence of upper respiratory tract infections and the number of sick days was markedly reduced (Evans et al. 2019). Considering the findings of these studies, the notable improvement in cold symptoms observed with *E. gracilis* intake in the present study may be, in part, attributed to the immunoregulatory action of paramylon. However, hot water extracts of *Euglena* also exhibit anti‐influenza virus activity in vitro, suggesting that the contribution of whole *Euglen*a should also be considered (Nakashima, Horio, et al. 2021).

The intake of *E. gracilis* reportedly regulates the autonomic nervous system balance under workload, improves irritability and tension under workload, and enhances sleep quality (Nakashima et al. 2020). Therefore, in the present study, we assessed subjective symptoms, such as physical fatigue, mental fatigue, and tension, using the VAS. However, we observed no significant differences between the groups. Our findings showed no significant differences in well‐being indicators between the groups, which could be attributed to the fact that participants were not selected based on baseline impairments in these domains. Moreover, the limited frequency of VAS assessments (only at weeks 0, 4, and 8) could have contributed to the reduced sensitivity to detect transient changes during the intervention period. Nevertheless, comparisons within the *Euglena* group demonstrated that the values for tension and relaxation were significantly lower (i.e., improved) after 8 weeks of intake than those at baseline. This aligns with the findings of Nakashima et al. (2020), who reported that *E. gracilis* intake alleviates irritability and tension. In the present study, comparisons within the *Euglena* group also demonstrated that satisfaction with defecation and refreshment upon urination significantly improved after 8 weeks of intake compared to baseline. These findings are supported by a previous study reporting that 30 consecutive days of *E. gracilis* intake can significantly increase defecation frequency and volume by improving the gut microbiota in healthy participants aged 40–59 years (Nakashima, Sasaki, et al. 2021). Overall, our findings further support the ability of *E. gracilis* to improve defecation.

To the best of our knowledge, the present study, which is a clinical trial conducted in Japan, includes the largest number of participants and spans the widest age range among immune‐related clinical studies on *Euglena* intake conducted to date. The mechanism by which *E. gracilis* intake is hypothesized to improve immune function involves the binding of paramylon in *E. gracilis* to Dectin‐1, triggering the release of various cytokines, including IL‐12, IL‐10, and IL‐6, and stimulating cells, such as NK, T, and B cells. However, this study had some limitations. First, it lacked an assessment of immune cell activity and other indicators that could substantiate the hypothesized mechanism of action. Therefore, further research is required to clarify the speculative involvement of enhanced immune function and elucidate the underlying mechanisms. Second, as some data were collected through participant diaries, which rely on subjective self‐reporting, the potential for recall bias cannot be ruled out. Third, because the analyses of secondary outcomes were exploratory, no adjustments for multiple comparisons were performed. Future confirmatory studies should incorporate such adjustments to account for potential Type I errors. Future studies are also warranted to gain insights into definitive evidence which may further clarify the mechanism of action of *Euglena* by measuring the activity of immune‐related cells and evaluating their interactions with various β‐glucan receptors.

## 5 Conclusions

The present study demonstrated that *E. gracilis* intake significantly alleviated cold symptoms by regulating immune function. This clinical trial, conducted in Japan, involved the widest age range and the largest number of participants among immune‐related clinical studies on *Euglena* intake. The findings also support the safety and efficacy of *E. gracilis* as a functional food. However, further studies evaluating immune cell activity are needed to confirm its mode of action.

## Author Contributions

**Ayaka Nakashima:** conceptualization (equal), formal analysis (equal), methodology (equal), resources (equal), writing – original draft (lead), writing – review and editing (equal). **Kengo Suzuki:** resources (equal), supervision (equal), writing – review and editing (equal). **Masafumi Nagata:** conceptualization (equal), writing – review and editing (supporting). **Tsuyoshi Takara:** conceptualization (equal), methodology (equal), supervision (equal), writing – review and editing (equal).

## Ethics Statement

This study was conducted in accordance with the tenets of the Declaration of Helsinki and the Ethical Guidelines for Medical and Health Research Involving Human Subjects. This study was approved by the Institutional Review Board of Takara Clinic (approval no. 2112‐00395: 2112‐00395‐0022‐1C‐TC, approval date: 12/22/2021).

## Consent

Written informed consent was obtained from all participants involved in the study.

## Conflicts of Interest

A.N. and K.S. are salaried employees of Euglena Co. Ltd., which produced some of the *Euglena* used in this study. All research funding for this study was provided by Euglena Co. Ltd. M.N. and T.T. were the medical advisors for this study and received honoraria from Euglena Co. Ltd. There are no other conflicts of interest.

## Supporting information


**Table S1:** Baseline data of the participants (cedar‐specific IgE).
**Table S2:** Baseline data of the participants (cypress‐specific IgE).
**Table S3:** Cold symptoms during the 5 days before baseline testing.
**Table S4:** Summary of primary safety assessment items.
**Table S5:** Summary of secondary safety assessment items.
**Table S6:** The highest number of consecutive days of cold symptoms overall and for individual symptoms.
**Table S7:** Proportions of responses regarding symptom severity.
**Table S8:** Well‐being factors measured with VAS.
**Table S9:** Well‐being factors measured with VAS (comparisons within the placebo group).
**Table S10:** Well‐being factors measured with VAS (comparisons within the *Euglena* group).

## Data Availability

Data supporting the results of this study are included in the Supporting Information to this paper. If further data are required, they can be provided upon reasonable request to the authors.

## References

[fsn370935-bib-0001] Bramley, T. J. , D. Lerner , and M. Sames . 2002. “Productivity Losses Related to the Common Cold.” Journal of Occupational and Environmental Medicine 44: 822–829. 10.1097/00043764-200209000-00004. 12227674

[fsn370935-bib-0002] Brauning, A. , M. Rae , G. Zhu , et al. 2022. “Aging of the Immune System: Focus on Natural Killer Cells Phenotype and Functions.” Cells 11: 1017. 10.3390/cells11061017. 35326467 PMC8947539

[fsn370935-bib-0003] Brown, G. D. , and S. Gordon . 2001. “A New Receptor for β‐Glucans.” Nature 413: 36–37. 10.1038/35092620. 11544516

[fsn370935-bib-0004] Cabanas, H. , K. Muraki , C. Balinas , N. Eaton‐Fitch , D. Staines , and S. Marshall‐Gradisnik . 2019. “Validation of Impaired Transient Receptor Potential Melastatin 3 Ion Channel Activity in Natural Killer Cells From Chronic Fatigue Syndrome/Myalgic Encephalomyelitis Patients.” Molecular Medicine 25: 14. 10.1186/s10020-019-0083-4. 31014226 PMC6480905

[fsn370935-bib-0005] Chan, G. C. F. , W. K. Chan , and D. M. Y. Sze . 2009. “The Effects of β‐Glucan on Human Immune and Cancer Cells.” Journal of Hematology & Oncology 2: 25. 10.1186/1756-8722-2-25. 19515245 PMC2704234

[fsn370935-bib-0006] Cohen‐Kedar, S. , L. Baram , H. Elad , E. Brazowski , H. Guzner‐Gur , and I. Dotan . 2014. “Human Intestinal Epithelial Cells Respond to β‐Glucans via Dectin‐1 and Syk.” European Journal of Immunology 44: 3729–3740. 10.1002/eji.201444876. 25251945

[fsn370935-bib-0007] Dai, J. , J. He , Z. Chen , et al. 2022. “ *Euglena gracilis* Promotes *Lactobacillus* Growth and Antioxidants Accumulation as a Potential Next‐Generation Prebiotic.” Frontiers in Nutrition 9: 864565. 10.3389/fnut.2022.864565. 35811960 PMC9257220

[fsn370935-bib-0009] Eccles, R. 2005. “Understanding the Symptoms of the Common Cold and Influenza.” Lancet Infectious Diseases 5: 718–725. 10.1016/S1473-3099(05)70270-X. 16253889 PMC7185637

[fsn370935-bib-0008] Eccles, R. 2009. “Mechanisms of Symptoms of Common Cold and Flu.” In Common Cold, edited by R. Eccles and O. Weber , 23–45. Birkhäuser Verlag. 10.1007/978-3-7643-9912-2_2.

[fsn370935-bib-0010] Evans, M. , P. H. Falcone , D. C. Crowley , et al. 2019. “Effect of a *Euglena Gracilis* Fermentate on Immune Function in Healthy, Active Adults: A Randomized, Double‐Blind, Placebo‐Controlled Trial.” Nutrients 11: 2926. 10.3390/nu11122926. 31816842 PMC6950611

[fsn370935-bib-0011] Ishibashi, K. I. , M. Nishioka , N. Onaka , et al. 2019. “Effects of *Euglena gracilis* EOD‐1 Ingestion on Salivary IgA Reactivity and Health‐Related Quality of Life in Humans.” Nutrients 11: 1144. 10.3390/nu11051144. 31121913 PMC6566313

[fsn370935-bib-0012] Kaufmann, S. H. E. 2019. “Immunology's Coming of Age.” Frontiers in Immunology 10, no. 1: 684. 10.3389/fimmu.2019.00684. 31001278 PMC6456699

[fsn370935-bib-0013] Kawano, T. , A. Miura , J. Naito , et al. 2023. “High‐Parameter Immune Profiling and Subjective Health Assessment of the Immunomodulatory Effects of Paramylon‐Rich *Euglena gracilis* EOD‐1: A Randomized, Double‐Blind, Placebo‐Controlled, Parallel‐Group Study.” Journal of Functional Foods 109: 105804. 10.1016/j.jff.2023.105804.

[fsn370935-bib-0014] Komastu, T. , D. D. Ireland , and C. S. Reiss . 1998. “IL‐12 and Viral Infections.” Cytokine & Growth Factor Reviews 9: 277–285. 10.1016/s1359-6101(98)00017-3. 9918125 PMC7129962

[fsn370935-bib-0015] Mah, E. , V. N. Kaden , K. M. Kelley , and D. J. Liska . 2020. “Beverage Containing Dispersible Yeast β‐Glucan Decreases Cold/Flu Symptomatic Days After Intense Exercise: A Randomized Controlled Trial.” Journal of Dietary Supplements 17, no. 2: 200–210. 10.1080/19390211.2018.1495676. 30380356

[fsn370935-bib-0016] Nakashima, A. , Y. Horio , K. Suzuki , and Y. Isegawa . 2021. “Antiviral Activity and Underlying Action Mechanism of Euglena Extract Against Influenza Virus.” Nutrients 13: 3911. 10.3390/nu13113911. 34836165 PMC8624635

[fsn370935-bib-0017] Nakashima, A. , K. Sasaki , D. Sasaki , K. Yasuda , K. Suzuki , and A. Kondo . 2021. “The Alga *Euglena gracilis* Stimulates Faecalibacterium in the Gut and Contributes to Increased Defecation.” Scientific Reports 11: 1074. 10.1038/s41598-020-80306-0. 33441865 PMC7806897

[fsn370935-bib-0018] Nakashima, A. , K. Suzuki , Y. Asayama , et al. 2017. “Oral Administration of *Euglena gracilis* Z and Its Carbohydrate Storage Substance Provides Survival Protection Against Influenza Virus Infection in Mice.” Biochemical and Biophysical Research Communications 494: 379–383. 10.1016/j.bbrc.2017.09.167. 28974421

[fsn370935-bib-0019] Nakashima, A. , K. Yamada , O. Iwata , et al. 2018. “β‐Glucan in Foods and Its Physiological Functions.” Journal of Nutritional Science and Vitaminology (Tokyo) 64: 8–17. 10.3177/jnsv.64.8. 29491277

[fsn370935-bib-0020] Nakashima, A. , K. Yasuda , A. Murata , K. Suzuki , and N. Miura . 2020. “Effects of *Euglena gracilis* Intake on Mood and Autonomic Activity Under Mental Workload, and Subjective Sleep Quality: A Randomized, Double‐Blind, Placebo‐Controlled Trial.” Nutrients 12: 3243. 10.3390/nu12113243. 33113956 PMC7690740

[fsn370935-bib-0021] Nguyen, T. , S. Johnston , L. Clarke , P. Smith , D. Staines , and S. Marshall‐Gradisnik . 2017. “Impaired Calcium Mobilization in Natural Killer Cells From Chronic Fatigue Syndrome/Myalgic Encephalomyelitis Patients Is Associated With Transient Receptor Potential Melastatin 3 Ion Channels.” Clinical and Experimental Immunology 187: 284–293. 10.1111/cei.12882. 27727448 PMC5217865

[fsn370935-bib-0022] Nguyen, T. , D. Staines , B. Nilius , P. Smith , and S. Marshall‐Gradisnik . 2016. “Novel Identification and Characterisation of Transient Receptor Potential Melastatin 3 Ion Channels on Natural Killer Cells and B Lymphocytes: Effects on Cell Signalling in Chronic Fatigue Syndrome/Myalgic Encephalomyelitis Patients.” Biological Research 49: 27. 10.1186/s40659-016-0087-2. 27245705 PMC4888729

[fsn370935-bib-0023] Ouyang, W. , and A. O'Garra . 2019. “IL‐10 Family Cytokines IL‐10 and IL‐22: From Basic Science to Clinical Translation.” Immunity 50: 871–891. 10.1016/j.immuni.2019.03.020. 30995504

[fsn370935-bib-0024] Papacosta, E. , and G. P. Nassis . 2011. “Saliva as a Tool for Monitoring Steroid, Peptide and Immune Markers in Sport and Exercise Science.” Journal of Science and Medicine in Sport 14: 424–434. 10.1016/j.jsams.2011.03.004. 21474377

[fsn370935-bib-0025] Park, S. Y. , K. J. Kim , S. M. Jo , et al. 2023. “ *Euglena gracilis* Powder Supplementation Enhanced Immune Function Through Natural Killer Cell Activity in Apparently Healthy Participants: A Randomized, Double‐Blind, Placebo‐Controlled Trial.” Nutrition Research 119: 90–97. 10.1016/j.nutres.2023.09.004. 37769481

[fsn370935-bib-0026] Piccirillo, G. , Q. Raffaele , F. Fimognari , et al. 2004. “Influence of L‐Arginine and Vitamin C on the Autonomic Nervous System in Chronic Heart Failure Secondary to Ischemic Cardiomyopathy.” American Journal of Cardiology 93: 650–654. 10.1016/j.amjcard.2003.11.043. 14996603

[fsn370935-bib-0027] Seiler, A. , C. P. Fagundes , and L. M. Christian . 2020. “The Impact of Everyday Stressors on the Immune System and Health.” In Stress Challenges and Immunity in Space, edited by A. Choukèr , 71–92. Springer International Publishing. 10.1007/978-3-030-16996-1_6.

[fsn370935-bib-0028] Shimada, R. , M. Fujita , M. Yuasa , et al. 2016. “Oral Administration of Green Algae, *Euglena gracilis* , Inhibits Hyperglycemia in OLETF Rats, a Model of Spontaneous Type 2 Diabetes.” Food & Function 7, no. 11: 4655–4659. 10.1039/c6fo00606j. 27775129

[fsn370935-bib-0029] Sugiyama, A. , S. Hata , K. Suzuki , et al. 2010. “Oral Administration of Paramylon, a β‐1,3‐D‐Glucan Isolated From *Euglena gracilis* Z Inhibits Development of Atopic Dermatitis‐Like Skin Lesions in NC/NGA Mice.” Journal of Veterinary Medical Science 72: 755–763. 10.1292/jvms.09-0526. 20160419

[fsn370935-bib-0030] Sugiyama, A. , K. Suzuki , S. Mitra , et al. 2009. “Hepatoprotective Effects of Paramylon, a BETA. 1,3‐D‐Glucan Isolated From *Euglena gracilis* Z, on Acute Liver Injury Induced by Carbon Tetrachloride in Rats.” Journal of Veterinary Medical Science 71: 885–890. 10.1292/jvms.71.885. 19652474

[fsn370935-bib-0031] Suzuki, K. 2017. “Large‐Scale Cultivation of Euglena.” Advances in Experimental Medicine and Biology 979: 285–293. 10.1007/978-3-319-54910-1_14. 28429327

[fsn370935-bib-0032] Tanaka, T. , M. Narazaki , and T. Kishimoto . 2014. “IL‐6 in Inflammation, Immunity, and Disease.” Cold Spring Harbor Perspectives in Biology 6: a016295. 10.1101/cshperspect.a016295. 25190079 PMC4176007

[fsn370935-bib-0033] Tirelli, U. , A. Lleshi , M. Berretta , M. Spina , R. Talamini , and A. Giacalone . 2013. “Treatment of 741 Italian Patients With Chronic Fatigue Syndrome.” European Review for Medical and Pharmacological Sciences 17: 2847–2852. 24254550

[fsn370935-bib-0034] Ujita, M. , H. Nagayama , S. Kanie , et al. 2009. “Carbohydrate Binding Specificity of Recombinant Human Macrophage β‐Glucan Receptor Dectin‐1.” Bioscience, Biotechnology, and Biochemistry 73: 237–240. 10.1271/bbb.80503. 19129647

[fsn370935-bib-0035] Wine, T. M. , and C. M. Alper . 2012. “Cytokine Responses in the Common Cold and Otitis Media.” Current Allergy and Asthma Reports 12: 574–581. 10.1007/s11882-012-0306-z. 23054624 PMC7089162

[fsn370935-bib-0036] Yasuda, K. , A. Nakashima , A. Murata , K. Suzuki , and T. Adachi . 2020. “ *Euglena gracilis* and β‐Glucan Paramylon Induce Ca2+ Signaling in Intestinal Tract Epithelial, Immune, and Neural Cells.” Nutrients 12: 2293. 10.3390/nu12082293. 32751743 PMC7468862

