# Supplementary material for: Euglena gracilis Suppresses Cold Symptoms in Healthy Individuals: A Double‐Blind, Randomized, Placebo‐Controlled Trial
Source: Food Sci Nutr. 2025 Sep 18;13(9):e70935. doi: 10.1002/fsn3.70935 (PMC12445114; doi:10.1002/fsn3.70935)
Supplement: Supplementary file 1 — Table S1: Baseline data of the participants (cedar‐specific IgE). Table S2: Baseline data of the participants (cypress‐specific IgE). Table S3: Cold symptoms during the 5 days before baseline testing. Table S4: Summary of primary safety assessment items. Table S5: Summary of secondary safety assessment items. Table S6: The highest number of consecutive days of cold symptoms overall and for individual symptoms. Table S7: Proportions of responses regarding symptom severity. Table S8: Well‐being factors measured with VAS. Table S9: Well‐being factors measured with VAS (comparisons within the placebo group). Table S10: Well‐being factors measured with VAS (comparisons within the Euglena group). [file FSN3-13-e70935-s001.docx]

Supplementary Information for

***Euglena gracilis* Suppresses Cold Symptoms in Healthy Individuals: A Double-Blind, Randomized, Placebo-Controlled Trial**

**Table S1.** Baseline data of the participants (cedar-specific IgE).

| **Item** | **Unit** |  |  | **Placebo group** | ***Euglena* group** |  |
| --- | --- | --- | --- | --- | --- | --- |
| Cases with cedar-specific IgE class 0 | - | n |  | 107 | 106 |  |
|  |  | Applicable |  | 42 (39.3%) | 40 (37.7%) |  |
|  |  | Not applicable |  | 65 (60.7%) | 66 (62.3%) |  |
|  |  | *P*-value (vs. Placebo group) * | | - | 0.888 |  |
| Cases with cedar-specific IgE class 1 | - | n |  | 107 | 106 |  |
|  |  | Applicable |  | 5 (4.7%) | 8 (7.5%) |  |
|  |  | Not applicable |  | 102 (95.3%) | 98 (92.5%) |  |
|  |  | *P*-value (vs. Placebo group) * |  | - | 0.408 |  |
| Cases with cedar-specific IgE class 2 | - | n |  | 107 | 106 |  |
|  |  | Applicable |  | 23 (21.5%) | 20 (18.9%) |  |
|  |  | Not applicable |  | 84 (78.5%) | 86 (81.1%) |  |
|  |  | *P*-value (vs. Placebo group) * | | - | 0.733 |  |
| Cases with cedar-specific IgE class 3 | - | n |  | 107 | 106 |  |
|  |  | Applicable |  | 25 (23.4%) | 33 (31.1%) |  |
|  |  | Not applicable |  | 82 (76.6%) | 73 (68.9%) |  |
|  |  | *P*-value (vs. Placebo group) * |  | - | 0.221 |  |
| Cases with cedar-specific IgE class 4 | - | n |  | 107 | 106 |  |
|  |  | Applicable |  | 12 (11.2%) | 5 (4.7%) |  |
|  |  | Not applicable |  | 95 (88.8%) | 101 (95.3%) |  |
|  |  | *P*-value (vs. Placebo group) * | | - | 0.127 |  |
| Cases with cedar-specific IgE class 5 | - | n |  | 107 | 106 |  |
|  |  | Applicable |  | 0 (0.0%) | 0 (0.0%) |  |
|  |  | Not applicable |  | 107 (100.0%) | 106 (100.0%) |  |
|  |  | *P*-value (vs. Placebo group) * |  | - | N.A. |  |
| Cases with cedar-specific IgE class 6 | - | n |  | 107 | 106 |  |
|  |  | Applicable |  | 0 (0.0%) | 0 (0.0%) |  |
|  |  | Not applicable |  | 107 (100.0%) | 106 (100.0%) |  |
|  |  | *P*-value (vs. Placebo group) * | | - | N.A. |  |
| Cedar-specific IgE | UA/mL | n |  | 107 | 106 |  |
|  |  | Mean (SD) |  | 5.2 (8.6) | 3.9 (6.1) |  |
|  |  | Med |  | 1.6 | 1.0 |  |
|  |  | Min-Max |  | 0.00-36.40 | 0.00–35.20 |  |
|  |  | *P*-value (vs. Placebo group) # | | - | 0.221 |  |

Max, Maximum; Med, Median; Min, Minimum; SD, Standard deviation.

#Between-group comparison with chi-square test; *between-group comparison with Welch’s *t-*test; n, number of participants. N.A.: Not available

**Table S2.** Baseline data of the participants (cypress-specific IgE).

| **Item** | **Unit** |  |  | **Placebo group** | ***Euglena* group** |  |
| --- | --- | --- | --- | --- | --- | --- |
| Cases with cypress-specific IgE class 0 | - | n |  | 107 | 106 |  |
|  |  | Applicable |  | 60 (56.1%) | 61 (57.5%) |  |
|  |  | Not applicable |  | 47 (43.9%) | 45 (42.5%) |  |
|  |  | *P*-value (vs. Placebo group) * | | - | 0.890 |  |
| Cases with cypress-specific IgE class 1 | - | n |  | 107 | 106 |  |
|  |  | Applicable |  | 10 (9.3%) | 14 (13.2%) |  |
|  |  | Not applicable |  | 97 (90.7%) | 92 (86.8%) |  |
|  |  | *P*-value (vs. Placebo group) * |  | - | 0.395 |  |
| Cases with cypress-specific IgE class 2 | - | n |  | 107 | 106 |  |
|  |  | Applicable |  | 28 (26.2%) | 27 (25.5%) |  |
|  |  | Not applicable |  | 79 (73.8%) | 79 (74.5%) |  |
|  |  | *P*-value (vs. Placebo group) * | | - | 1.000 |  |
| Cases with cypress-specific IgE class 3 | - | n |  | 107 | 106 |  |
|  |  | Applicable |  | 9 (8.4%) | 4 (3.8%) |  |
|  |  | Not applicable |  | 98 (91.6%) | 102 (96.2%) |  |
|  |  | *P*-value (vs. Placebo group) * |  | - | 0.252 |  |
| Cases with cypress-specific IgE class 4 | - | n |  | 107 | 106 |  |
|  |  | Applicable |  | 0 (0.0%) | 0 (0.0%) |  |
|  |  | Not applicable |  | 107 (100.0%) | 106 (100.0%) |  |
|  |  | *P*-value (vs. Placebo group) * | | - | N.A. |  |
| Cases with cypress-specific IgE class 5 | - | n |  | 107 | 106 |  |
|  |  | Applicable |  | 0 (0.0%) | 0 (0.0%) |  |
|  |  | Not applicable |  | 107 (100.0%) | 106 (100.0%) |  |
|  |  | *P*-value (vs. Placebo group) * |  | - | N.A. |  |
| Cases with cypress-specific IgE class 6 | - | n |  | 107 | 106 |  |
|  |  | Applicable |  | 0 (0.0%) | 0 (0.0%) |  |
|  |  | Not applicable |  | 107 (100.0%) | 106 (100.0%) |  |
|  |  | *P*-value (vs .Placebo group) * | | - | N.A. |  |
| cypress-specific IgE | UA/mL | n |  | 107 | 106 |  |
|  |  | Mean (SD) |  | 1.1 (1.9) | 0.8 (1.4) |  |
|  |  | Med |  | 0.2 | 0.2 |  |
|  |  | Min-Max |  | 0.00–9.42 | 0.00–9.31 |  |
|  |  | *P*-value (vs. Placebo group) # | | - | 0.207 |  |

Max, Maximum; Med, Median; Min, Minimum; SD, Standard deviation.

#Between-group com-parison with chi-square test; *between-group comparison with Welch’s *t*-test

n, number of participants. N.A.: Not available

**Table S3**. Cold symptoms during the five days before baseline testing.

| **Item** | **Period** | **Unit** | **Placebo group** | | | | | |  | ***Euglena* group** | | | | | |  | **Between-group comparisons** | | | | |
| --- | --- | --- | --- | --- | --- | --- | --- | --- | --- | --- | --- | --- | --- | --- | --- | --- | --- | --- | --- | --- | --- |
|  |  |  | n | Mean | SD | Med | Min | Max |  | n | Mean | SD | Med | Min | Max |  | ⊿ | SE | 95% CI- | 95% CI+ | *P*-value |
| Days with cold symptoms | Last 5 days | days | 107 | 2.5 | 1.7 | 2.0 | 1.0 | 5.0 |  | 106 | 2.6 | 1.7 | 2.0 | 1.0 | 5.0 |  | 0.1 | 0.2 | -0.4 | 0.5 | 0.732 |
| Days with general malaise | Last 5 days | days | 107 | 0.8 | 1.4 | 0.0 | 0.0 | 5.0 |  | 106 | 0.7 | 1.5 | 0.0 | 0.0 | 5.0 |  | 0.0 | 0.2 | -0.5 | 0.4 | 0.809 |
| Days with chills | Last 5 days | days | 107 | 0.3 | 1.0 | 0.0 | 0.0 | 5.0 |  | 106 | 0.3 | 1.0 | 0.0 | 0.0 | 5.0 |  | 0.0 | 0.1 | -0.3 | 0.3 | 0.965 |
| Days with feverishness | Last 5 days | days | 107 | 0.1 | 0.5 | 0.0 | 0.0 | 4.0 |  | 106 | 0.1 | 0.7 | 0.0 | 0.0 | 5.0 |  | 0.0 | 0.1 | -0.2 | 0.1 | 0.826 |
| Days with fatigue | Last 5 days | days | 107 | 1.8 | 1.8 | 1.0 | 0.0 | 5.0 |  | 106 | 1.8 | 1.8 | 1.0 | 0.0 | 5.0 |  | 0.0 | 0.2 | -0.4 | 0.5 | 0.856 |
| Days with sneezing | Last 5 days | days | 107 | 0.3 | 1.0 | 0.0 | 0.0 | 5.0 |  | 106 | 0.3 | 0.9 | 0.0 | 0.0 | 5.0 |  | 0.0 | 0.1 | -0.2 | 0.3 | 0.927 |
| Days with runny nose | Last 5 days | days | 107 | 0.6 | 1.3 | 0.0 | 0.0 | 5.0 |  | 106 | 0.5 | 1.3 | 0.0 | 0.0 | 5.0 |  | 0.0 | 0.2 | -0.4 | 0.3 | 0.941 |
| Days with nasal congestion | Last 5 days | days | 107 | 0.2 | 0.9 | 0.0 | 0.0 | 5.0 |  | 106 | 0.2 | 0.8 | 0.0 | 0.0 | 5.0 |  | 0.0 | 0.1 | -0.2 | 0.2 | 0.986 |
| Days with sore throat | Last 5 days | days | 107 | 0.1 | 0.3 | 0.0 | 0.0 | 2.0 |  | 106 | 0.1 | 0.5 | 0.0 | 0.0 | 4.0 |  | 0.0 | 0.1 | -0.1 | 0.1 | 0.711 |
| Days with coughing | Last 5 days | days | 107 | 0.0 | 0.2 | 0.0 | 0.0 | 2.0 |  | 106 | 0.1 | 0.4 | 0.0 | 0.0 | 3.0 |  | 0.0 | 0.0 | 0.0 | 0.1 | 0.383 |
| Days with arthralgia | Last 5 days | days | 107 | 0.4 | 1.2 | 0.0 | 0.0 | 5.0 |  | 106 | 0.3 | 1.0 | 0.0 | 0.0 | 5.0 |  | 0.0 | 0.2 | -0.3 | 0.3 | 0.778 |
| Days with myalgia | Last 5 days | days | 107 | 0.3 | 0.9 | 0.0 | 0.0 | 5.0 |  | 106 | 0.4 | 1.1 | 0.0 | 0.0 | 5.0 |  | 0.1 | 0.1 | -0.2 | 0.4 | 0.442 |
| Days with headache | Last 5 days | days | 107 | 0.3 | 0.8 | 0.0 | 0.0 | 4.0 |  | 106 | 0.2 | 0.7 | 0.0 | 0.0 | 5.0 |  | 0.0 | 0.1 | -0.2 | 0.2 | 0.734 |
| Severity of general malaise | Last 5 days | - | 107 | 1.5 | 0.8 | 1.0 | 1.0 | 4.0 |  | 106 | 1.5 | 0.9 | 1.0 | 1.0 | 5.0 |  | 0.0 | 0.1 | -0.2 | 0.3 | 0.728 |
| Severity of chills | Last 5 days | - | 107 | 1.2 | 0.6 | 1.0 | 1.0 | 4.0 |  | 106 | 1.3 | 0.6 | 1.0 | 1.0 | 4.0 |  | 0.0 | 0.1 | -0.1 | 0.2 | 0.713 |
| Severity of feverishness | Last 5 days | - | 107 | 1.1 | 0.3 | 1.0 | 1.0 | 3.0 |  | 106 | 1.1 | 0.5 | 1.0 | 1.0 | 4.0 |  | 0.0 | 0.1 | -0.1 | 0.1 | 0.592 |
| Severity of fatigue | Last 5 days | - | 107 | 2.0 | 1.0 | 2.0 | 1.0 | 5.0 |  | 106 | 2.0 | 1.1 | 2.0 | 1.0 | 5.0 |  | 0.1 | 0.1 | -0.2 | 0.3 | 0.703 |
| Severity of sneezing | Last 5 days | - | 107 | 1.2 | 0.5 | 1.0 | 1.0 | 3.0 |  | 106 | 1.3 | 0.5 | 1.0 | 1.0 | 3.0 |  | 0.0 | 0.1 | -0.1 | 0.2 | 0.872 |
| Severity of runny nose | Last 5 days | - | 107 | 1.4 | 0.7 | 1.0 | 1.0 | 4.0 |  | 106 | 1.4 | 0.7 | 1.0 | 1.0 | 4.0 |  | 0.0 | 0.1 | -0.1 | 0.2 | 0.670 |
| Severity of nasal congestion | Last 5 days | - | 107 | 1.2 | 0.5 | 1.0 | 1.0 | 4.0 |  | 106 | 1.2 | 0.5 | 1.0 | 1.0 | 3.0 |  | 0.0 | 0.1 | -0.1 | 0.2 | 0.666 |
| Severity of sore throat | Last 5 days | - | 107 | 1.1 | 0.3 | 1.0 | 1.0 | 2.0 |  | 106 | 1.1 | 0.4 | 1.0 | 1.0 | 3.0 |  | 0.0 | 0.0 | -0.1 | 0.1 | 0.821 |
| Severity of coughing | Last 5 days | - | 107 | 1.1 | 0.3 | 1.0 | 1.0 | 2.0 |  | 106 | 1.1 | 0.3 | 1.0 | 1.0 | 3.0 |  | 0.0 | 0.0 | -0.1 | 0.1 | 0.852 |
| Severity of arthralgia | Last 5 days | - | 107 | 1.2 | 0.6 | 1.0 | 1.0 | 4.0 |  | 106 | 1.3 | 0.6 | 1.0 | 1.0 | 4.0 |  | 0.0 | 0.1 | -0.1 | 0.2 | 0.805 |
| Severity of myalgia | Last 5 days | - | 107 | 1.2 | 0.6 | 1.0 | 1.0 | 4.0 |  | 106 | 1.3 | 0.7 | 1.0 | 1.0 | 5.0 |  | 0.1 | 0.1 | -0.1 | 0.2 | 0.432 |
| Severity of headache | Last 5 days | - | 107 | 1.2 | 0.5 | 1.0 | 1.0 | 4.0 |  | 106 | 1.2 | 0.5 | 1.0 | 1.0 | 4.0 |  | 0.0 | 0.1 | -0.1 | 0.2 | 0.879 |

n, Number of participants; Max, Maximum; Med, Median; Min, Minimum; SD, Standard deviation; SE, Standard error. Between-group comparisons were performed with Welch’s *t*-test; ⊿, Difference between groups (*Euglena* group versus placebo group); n, number of participants; 95% CI, lower limit of 95% confidence interval; 95% CI+, upper limit of 95% confidence interval.

**Table S4.** Summary of primary safety assessment items.

| **Item** | **Placebo group** | | |  | ***Euglena* group** | | |  | **Between-group comparisons** | | | | |
| --- | --- | --- | --- | --- | --- | --- | --- | --- | --- | --- | --- | --- | --- |
|  | **n** | **No. of cases** | **Incidence (%)** |  | **n** | **No. of cases** | **Incidence (%)** |  | ⊿ **(%)** | **95% CI−** | **95% CI+** | **χ^2^** | ***P*-value** |
| Incidence of adverse drug reactions | 107 | 0 | 0.0 |  | 106 | 0 | 0.0 |  | 0.0 | N.A. | N.A. | N.A. | N.A. |
| Incidence of adverse events | 107 | 18 | 16.8 |  | 106 | 15 | 14.2 |  | -2.7 | -12.4 | 7.0 | 0.290 | 0.705 |

n, Number of participants; Between-group comparisons with chi-square test; N.A., Not available; ⊿, Difference in incidence (*Euglena* group versus placebo group); 95% CI−, Lower bound of 95% confidence interval of incidence difference; 95% CI+, Upper bound of 95% confidence interval.

**Table S5.** Summary of secondary safety assessment items.

| Item | Time | **Placebo group** | | |  | ***Euglena* group** | | |  | **Between-group comparisons** | | | | |
| --- | --- | --- | --- | --- | --- | --- | --- | --- | --- | --- | --- | --- | --- | --- |
|  |  | n | No. of applicable cases | Rate of applicable cases (%) |  | n | No. of cases | Incidence (%) |  | ⊿ (%) | 95% CI− | 95% CI+ | χ^2^ | *P*-value |
| Urine protein | Week 8 | 107 | 4 | 3.7 |  | 106 | 0 | 0.0 |  | -3.7 | -7.4 | -0.1 | 4.038 | 0.121 |
| Urine glucose | Week 8 | 107 | 2 | 1.9 |  | 106 | 0 | 0.0 |  | -1.9 | -4.5 | 0.7 | 2.000 | 0.498 |
| Urine pH | Week 8 | 107 | 0 | 0.0 |  | 106 | 2 | 1.9 |  | 1.9 | -0.7 | 4.5 | 2.038 | 0.246 |
| Urine occult blood | Week 8 | 107 | 2 | 1.9 |  | 106 | 4 | 3.8 |  | 1.9 | -2.5 | 6.3 | 0.705 | 0.445 |
| White blood cell count | Week 8 | 107 | 4 | 3.7 |  | 106 | 1 | 0.9 |  | -2.8 | -6.9 | 1.3 | 1.815 | 0.369 |
| Red blood cell count | Week 8 | 107 | 2 | 1.9 |  | 106 | 3 | 2.8 |  | 1.0 | -3.1 | 5.0 | 0.215 | 0.683 |
| Hemoglobin | Week 8 | 107 | 2 | 1.9 |  | 106 | 2 | 1.9 |  | 0.0 | -3.6 | 3.7 | 0.000 | 1.000 |
| Hematocrit | Week 8 | 107 | 4 | 3.7 |  | 106 | 2 | 1.9 |  | -1.9 | -6.3 | 2.6 | 0.667 | 0.683 |
| Platelet count | Week 8 | 107 | 2 | 1.9 |  | 106 | 7 | 6.6 |  | 4.7 | -0.7 | 10.1 | 2.950 | 0.101 |
| AST (GOT) | Week 8 | 107 | 1 | 0.9 |  | 106 | 2 | 1.9 |  | 1.0 | -2.2 | 4.1 | 0.348 | 0.621 |
| ALT (GPT) | Week 8 | 107 | 0 | 0.0 |  | 106 | 0 | 0.0 |  | 0.0 | N.A. | N.A. | N.A. | N.A. |
| γ-GT (γ-GTP) | Week 8 | 107 | 0 | 0.0 |  | 106 | 3 | 2.8 |  | 2.8 | -0.3 | 6.0 | 3.072 | 0.121 |
| Total bilirubin | Week 8 | 107 | 0 | 0.0 |  | 106 | 1 | 0.9 |  | 0.9 | -0.9 | 2.8 | 1.014 | 0.498 |
| Total protein | Week 8 | 107 | 9 | 8.4 |  | 106 | 4 | 3.8 |  | -4.6 | -11.1 | 1.8 | 1.998 | 0.252 |
| Urea nitrogen | Week 8 | 107 | 2 | 1.9 |  | 106 | 5 | 4.7 |  | 2.8 | -1.9 | 7.6 | 1.359 | 0.280 |
| Creatinine | Week 8 | 107 | 3 | 2.8 |  | 106 | 3 | 2.8 |  | 0.0 | -4.4 | 4.5 | 0.000 | 1.000 |
| Uric acid | Week 8 | 107 | 2 | 1.9 |  | 106 | 3 | 2.8 |  | 1.0 | -3.1 | 5.0 | 0.215 | 0.683 |
| Sodium | Week 8 | 107 | 0 | 0.0 |  | 106 | 1 | 0.9 |  | 0.9 | -0.9 | 2.8 | 1.014 | 0.498 |
| Potassium | Week 8 | 107 | 7 | 6.5 |  | 106 | 4 | 3.8 |  | -2.8 | -8.7 | 3.2 | 0.833 | 0.538 |
| Chloride | Week 8 | 107 | 0 | 0.0 |  | 106 | 1 | 0.9 |  | 0.9 | -0.9 | 2.8 | 1.014 | 0.498 |
| Serum amylase | Week 8 | 107 | 1 | 0.9 |  | 106 | 3 | 2.8 |  | 1.9 | -1.8 | 5.5 | 1.038 | 0.369 |
| Total cholesterol | Week 8 | 107 | 7 | 6.5 |  | 106 | 7 | 6.6 |  | 0.1 | -6.6 | 6.7 | 0.000 | 1.000 |
| HDL cholesterol | Week 8 | 107 | 5 | 4.7 |  | 106 | 3 | 2.8 |  | -1.8 | -6.9 | 3.3 | 0.500 | 0.721 |
| LDL cholesterol | Week 8 | 107 | 4 | 3.7 |  | 106 | 6 | 5.7 |  | 1.9 | -3.8 | 7.6 | 0.440 | 0.538 |
| Triglycerides | Week 8 | 107 | 6 | 5.6 |  | 106 | 4 | 3.8 |  | -1.8 | -7.5 | 3.8 | 0.400 | 0.748 |
| Glucose | Week 8 | 107 | 3 | 2.8 |  | 106 | 4 | 3.8 |  | 1.0 | -3.8 | 5.8 | 0.158 | 0.721 |
| HbA1c (NGSP) | Week 8 | 107 | 0 | 0.0 |  | 106 | 0 | 0.0 |  | 0.0 | N.A. | N.A. | N.A. | N.A. |

n, Number of participants; ⊿, Difference in rate of applicable cases (*Euglena* group versus placebo group); 95% CI−, Lower bound of 95% confidence interval for incidence difference; 95% CI+, Upper bound of 95% confidence interval.

Between-group comparisons were performed with Chi-square test; N.A., Not available. No. proportion of cases in which the urinary and peripheral blood test values were within the reference range at the time of screening and pre-intake examination shifted outside the reference range after intervention.

**Table S6.** The highest number of consecutive days of cold symptoms overall and for individual symptoms.

| **Item** | **Period** | **Unit** | **Placebo group** | | | | | |  | | | ***Euglena* group** | | | | | |  | | | **Between-group comparisons** | | | | |
| --- | --- | --- | --- | --- | --- | --- | --- | --- | --- | --- | --- | --- | --- | --- | --- | --- | --- | --- | --- | --- | --- | --- | --- | --- | --- |
|  |  |  | n | Mean | SD | Med | Min | Max | |  | n | | Mean | SD | Med | Min | Max | |  | ⊿ | | SE | 95% CI− | 95% CI+ | *P*-value |
| Highest number of consecutive days with cold symptoms | Overall | Days | 107 | 10.9 | 14.5 | 5.0 | 0.0 | 56.0 | |  | 106 | | 8.8 | 14.4 | 3.0 | 0.0 | 56.0 | |  | -2.1 | | 2.0 | -6.0 | 1.8 | 0.281 |
|  | 1st half | Days | 107 | 6.4 | 8.0 | 3.0 | 0.0 | 28.0 | |  | 106 | | 5.3 | 8.0 | 2.0 | 0.0 | 28.0 | |  | -1.1 | | 1.1 | -3.3 | 1.0 | 0.306 |
|  | 2nd half | Days | 107 | 7.3 | 9.3 | 4.0 | 0.0 | 28.0 | |  | 106 | | 5.2 | 8.3 | 2.0 | 0.0 | 28.0 | |  | -2.1 | | 1.2 | -4.4 | 0.3 | 0.087 |
| Highest number of consecutive days with general malaise | Overall | Days | 107 | 3.0 | 7.0 | 1.0 | 0.0 | 45.0 | |  | 106 | | 3.3 | 9.3 | 0.0 | 0.0 | 56.0 | |  | 0.3 | | 1.1 | -1.9 | 2.6 | 0.757 |
|  | 1st half | Days | 107 | 1.7 | 3.0 | 0.0 | 0.0 | 17.0 | |  | 106 | | 2.0 | 5.2 | 0.0 | 0.0 | 28.0 | |  | 0.4 | | 0.6 | -0.8 | 1.5 | 0.510 |
|  | 2nd half | Days | 107 | 2.1 | 5.1 | 0.0 | 0.0 | 28.0 | |  | 106 | | 1.7 | 4.9 | 0.0 | 0.0 | 28.0 | |  | -0.4 | | 0.7 | -1.8 | 0.9 | 0.535 |
| Highest number of consecutive days with chills | Overall | Days | 107 | 0.7 | 2.2 | 0.0 | 0.0 | 20.0 | |  | 106 | | 0.5 | 1.6 | 0.0 | 0.0 | 14.0 | |  | -0.2 | | 0.3 | -0.7 | 0.4 | 0.552 |
|  | 1st half | Days | 107 | 0.4 | 2.0 | 0.0 | 0.0 | 20.0 | |  | 106 | | 0.4 | 1.5 | 0.0 | 0.0 | 14.0 | |  | 0.0 | | 0.2 | -0.5 | 0.5 | 0.983 |
|  | 2nd half | Days | 107 | 0.4 | 1.0 | 0.0 | 0.0 | 6.0 | |  | 106 | | 0.2 | 0.5 | 0.0 | 0.0 | 3.0 | |  | -0.2 | | 0.1 | -0.4 | 0.0 | 0.084 |
| Highest number of consecutive days with feverishness | Overall | Days | 107 | 0.4 | 0.9 | 0.0 | 0.0 | 5.0 | |  | 106 | | 0.5 | 1.1 | 0.0 | 0.0 | 7.0 | |  | 0.1 | | 0.1 | -0.2 | 0.4 | 0.622 |
|  | 1st half | Days | 107 | 0.2 | 0.6 | 0.0 | 0.0 | 4.0 | |  | 106 | | 0.3 | 1.0 | 0.0 | 0.0 | 7.0 | |  | 0.1 | | 0.1 | -0.1 | 0.3 | 0.298 |
|  | 2nd half | Days | 107 | 0.4 | 0.9 | 0.0 | 0.0 | 5.0 | |  | 106 | | 0.3 | 0.8 | 0.0 | 0.0 | 5.0 | |  | -0.1 | | 0.1 | -0.3 | 0.1 | 0.385 |
| Highest number of consecutive days with fatigue | Overall | Days | 107 | 5.4 | 8.8 | 2.0 | 0.0 | 40.0 | |  | 106 | | 4.9 | 10.6 | 1.0 | 0.0 | 56.0 | |  | -0.5 | | 1.3 | -3.2 | 2.1 | 0.686 |
|  | 1st half | Days | 107 | 3.2 | 5.0 | 1.0 | 0.0 | 26.0 | |  | 106 | | 3.0 | 5.4 | 1.0 | 0.0 | 28.0 | |  | -0.2 | | 0.7 | -1.7 | 1.2 | 0.734 |
|  | 2nd half | Days | 107 | 4.1 | 7.0 | 1.0 | 0.0 | 28.0 | |  | 106 | | 3.0 | 6.4 | 1.0 | 0.0 | 28.0 | |  | -1.1 | | 0.9 | -2.9 | 0.7 | 0.220 |
| Highest number of consecutive days with sneezing | Overall | Days | 107 | 1.9 | 4.0 | 0.0 | 0.0 | 25.0 | |  | 106 | | 2.0 | 5.3 | 0.0 | 0.0 | 30.0 | |  | 0.1 | | 0.6 | -1.2 | 1.4 | 0.874 |
|  | 1st half | Days | 107 | 1.0 | 2.5 | 0.0 | 0.0 | 14.0 | |  | 106 | | 0.9 | 2.7 | 0.0 | 0.0 | 15.0 | |  | -0.1 | | 0.4 | -0.8 | 0.6 | 0.852 |
|  | 2nd half | Days | 107 | 1.4 | 3.2 | 0.0 | 0.0 | 19.0 | |  | 106 | | 1.3 | 4.0 | 0.0 | 0.0 | 24.0 | |  | 0.0 | | 0.5 | -1.0 | 0.9 | 0.945 |
| Highest number of consecutive days with runny nose | Overall | Days | 107 | 3.6 | 8.2 | 0.0 | 0.0 | 54.0 | |  | 106 | | 3.2 | 8.7 | 0.0 | 0.0 | 56.0 | |  | -0.4 | | 1.2 | -2.7 | 1.9 | 0.718 |
|  | 1st half | Days | 107 | 1.8 | 4.4 | 0.0 | 0.0 | 27.0 | |  | 106 | | 1.6 | 4.6 | 0.0 | 0.0 | 28.0 | |  | -0.2 | | 0.6 | -1.4 | 1.1 | 0.782 |
|  | 2nd half | Days | 107 | 2.5 | 6.0 | 0.0 | 0.0 | 28.0 | |  | 106 | | 1.9 | 5.2 | 0.0 | 0.0 | 28.0 | |  | -0.6 | | 0.8 | -2.1 | 0.9 | 0.415 |
| Highest number of consecutive days with nasal congestion | Overall | Days | 107 | 1.4 | 3.7 | 0.0 | 0.0 | 25.0 | |  | 106 | | 1.1 | 3.7 | 0.0 | 0.0 | 29.0 | |  | -0.3 | | 0.5 | -1.3 | 0.7 | 0.521 |
|  | 1st half | Days | 107 | 0.7 | 1.9 | 0.0 | 0.0 | 9.0 | |  | 106 | | 0.6 | 2.2 | 0.0 | 0.0 | 15.0 | |  | -0.1 | | 0.3 | -0.7 | 0.5 | 0.733 |
|  | 2nd half | Days | 107 | 1.1 | 3.2 | 0.0 | 0.0 | 19.0 | |  | 106 | | 0.5 | 2.2 | 0.0 | 0.0 | 14.0 | |  | -0.5 | | 0.4 | -1.3 | 0.2 | 0.148 |
| Highest number of consecutive days with sore throat | Overall | Days | 107 | 1.7 | 6.4 | 0.0 | 0.0 | 56.0 | |  | 106 | | 1.0 | 3.0 | 0.0 | 0.0 | 18.0 | |  | -0.8 | | 0.7 | -2.1 | 0.6 | 0.271 |
|  | 1st half | Days | 107 | 1.0 | 3.9 | 0.0 | 0.0 | 28.0 | |  | 106 | | 0.9 | 2.8 | 0.0 | 0.0 | 15.0 | |  | -0.2 | | 0.5 | -1.1 | 0.7 | 0.687 |
|  | 2nd half | Days | 107 | 0.9 | 3.3 | 0.0 | 0.0 | 28.0 | |  | 106 | | 0.4 | 1.5 | 0.0 | 0.0 | 13.0 | |  | -0.5 | | 0.3 | -1.2 | 0.2 | 0.185 |
| Highest number of consecutive days with coughing | Overall | Days | 107 | 1.5 | 6.1 | 0.0 | 0.0 | 56.0 | |  | 106 | | 0.7 | 2.2 | 0.0 | 0.0 | 16.0 | |  | -0.8 | | 0.6 | -2.0 | 0.5 | 0.230 |
|  | 1st half | Days | 107 | 0.8 | 3.3 | 0.0 | 0.0 | 28.0 | |  | 106 | | 0.5 | 1.5 | 0.0 | 0.0 | 10.0 | |  | -0.3 | | 0.4 | -1.0 | 0.4 | 0.333 |
|  | 2nd half | Days | 107 | 0.9 | 3.3 | 0.0 | 0.0 | 28.0 | |  | 106 | | 0.4 | 1.8 | 0.0 | 0.0 | 16.0 | |  | -0.5 | | 0.4 | -1.2 | 0.2 | 0.189 |
| Highest number of consecutive days with arthralgia | Overall | Days | 107 | 1.1 | 2.6 | 0.0 | 0.0 | 17.0 | |  | 106 | | 1.3 | 5.3 | 0.0 | 0.0 | 45.0 | |  | 0.2 | | 0.6 | -1.0 | 1.3 | 0.768 |
|  | 1st half | Days | 107 | 0.6 | 2.2 | 0.0 | 0.0 | 17.0 | |  | 106 | | 0.9 | 4.0 | 0.0 | 0.0 | 28.0 | |  | 0.3 | | 0.4 | -0.5 | 1.2 | 0.470 |
|  | 2nd half | Days | 107 | 0.7 | 1.8 | 0.0 | 0.0 | 12.0 | |  | 106 | | 0.7 | 3.0 | 0.0 | 0.0 | 25.0 | |  | 0.0 | | 0.3 | -0.6 | 0.7 | 0.941 |
| Highest number of consecutive days with myalgia | Overall | Days | 107 | 1.5 | 4.4 | 0.0 | 0.0 | 39.0 | |  | 106 | | 0.9 | 3.0 | 0.0 | 0.0 | 27.0 | |  | -0.6 | | 0.5 | -1.6 | 0.4 | 0.235 |
|  | 1st half | Days | 107 | 0.8 | 2.1 | 0.0 | 0.0 | 11.0 | |  | 106 | | 0.6 | 1.8 | 0.0 | 0.0 | 11.0 | |  | -0.1 | | 0.3 | -0.7 | 0.4 | 0.590 |
|  | 2nd half | Days | 107 | 1.1 | 3.5 | 0.0 | 0.0 | 28.0 | |  | 106 | | 0.5 | 2.1 | 0.0 | 0.0 | 20.0 | |  | -0.6 | | 0.4 | -1.4 | 0.1 | 0.106 |
| Highest number of consecutive days with headache | Overall | Days | 107 | 1.5 | 2.8 | 0.0 | 0.0 | 16.0 | |  | 106 | | 1.3 | 4.7 | 0.0 | 0.0 | 47.0 | |  | -0.2 | | 0.5 | -1.2 | 0.9 | 0.755 |
|  | 1st half | Days | 107 | 0.9 | 2.2 | 0.0 | 0.0 | 16.0 | |  | 106 | | 0.8 | 2.0 | 0.0 | 0.0 | 19.0 | |  | -0.1 | | 0.3 | -0.7 | 0.4 | 0.690 |
|  | 2nd half | Days | 107 | 1.0 | 1.8 | 0.0 | 0.0 | 12.0 | |  | 106 | | 0.8 | 3.0 | 0.0 | 0.0 | 28.0 | |  | -0.1 | | 0.3 | -0.8 | 0.5 | 0.717 |

Max, Maximum; Med, Median; Min, Minimum; SD, Standard deviation; SE, Standard error of the difference between groups. n, Number of participants; ⊿, Difference between groups (*Euglena* group versus placebo group); 95% CI−, Lower bound of 95% confidence interval; 95% CI+, Upper bound of 95% confidence interval; †, Primary outcome; Between-group comparisons were performed with Welch’s *t*-test, **P* < 0.05, ***P* < 0.01.

**Table S7.** Proportions of responses regarding symptom severity.

| **Item** | **Period** | **Placebo group** | | | | ***Euglena* group** | | | | **Between-group comparisons** | | | | | |
| --- | --- | --- | --- | --- | --- | --- | --- | --- | --- | --- | --- | --- | --- | --- | --- |
|  |  | Total participant days | Applicable days | Percentage of applicable days (%) | Total participant days | | Applicable days | Percentage of applicable days (%) | ⊿ (%) | | 95% CI- | 95% CI+ | χ^2^ | *P*-value |  |
| Percentage of severity 2 general malaise | Overall | 5992 | 1410 | 23.5 | 5936 | | 1335 | 22.5 | -1.0 | | -2.6 | 0.5 | 1.826 | 0.178 |  |
|  | 1st half | 2996 | 744 | 24.8 | 2968 | | 676 | 22.8 | -2.1 | | -4.2 | 0.1 | 3.477 | 0.064 |  |
|  | 2nd half | 2996 | 666 | 22.2 | 2968 | | 659 | 22.2 | 0.0 | | -2.1 | 2.1 | 0.001 | 1.000 |  |
| Percentage of severity 3 general malaise | Overall | 5992 | 467 | 7.8 | 5936 | | 448 | 7.5 | -0.2 | | -1.2 | 0.7 | 0.256 | 0.630 |  |
|  | 1st half | 2996 | 227 | 7.6 | 2968 | | 243 | 8.2 | 0.6 | | -0.8 | 2.0 | 0.766 | 0.387 |  |
|  | 2nd half | 2996 | 240 | 8.0 | 2968 | | 205 | 6.9 | -1.1 | | -2.4 | 0.2 | 2.630 | 0.115 |  |
| Percentage of severity 4 general malaise | Overall | 5992 | 129 | 2.2 | 5936 | | 123 | 2.1 | -0.1 | | -0.6 | 0.4 | 0.094 | 0.799 |  |
|  | 1st half | 2996 | 62 | 2.1 | 2968 | | 86 | 2.9 | 0.8 | | 0.0 | 1.6 | 4.225 | 0.045* |  |
|  | 2nd half | 2996 | 67 | 2.2 | 2968 | | 37 | 1.2 | -1.0 | | -1.7 | -0.3 | 8.523 | 0.004** |  |
| Percentage of severity 2 chills | Overall | 5992 | 750 | 12.5 | 5936 | | 535 | 9.0 | -3.5 | | -4.6 | -2.4 | 38.086 | 0.000*** |  |
|  | 1st half | 2996 | 403 | 13.5 | 2968 | | 320 | 10.8 | -2.7 | | -4.3 | -1.0 | 9.974 | 0.002** |  |
|  | 2nd half | 2996 | 347 | 11.6 | 2968 | | 215 | 7.2 | -4.3 | | -5.8 | -2.9 | 32.875 | 0.000*** |  |
| Percentage of severity 3 chills | Overall | 5992 | 99 | 1.7 | 5936 | | 78 | 1.3 | -0.3 | | -0.8 | 0.1 | 2.333 | 0.130 |  |
|  | 1st half | 2996 | 62 | 2.1 | 2968 | | 55 | 1.9 | -0.2 | | -0.9 | 0.5 | 0.363 | 0.576 |  |
|  | 2nd half | 2996 | 37 | 1.2 | 2968 | | 23 | 0.8 | -0.5 | | -1.0 | 0.0 | 3.168 | 0.091 |  |
| Percentage of severity 4 chills | Overall | 5992 | 23 | 0.4 | 5936 | | 22 | 0.4 | 0.0 | | -0.2 | 0.2 | 0.014 | 1.000 |  |
|  | 1st half | 2996 | 8 | 0.3 | 2968 | | 18 | 0.6 | 0.3 | | 0.0 | 0.7 | 3.958 | 0.051 |  |
|  | 2nd half | 2996 | 15 | 0.5 | 2968 | | 4 | 0.1 | -0.4 | | -0.7 | -0.1 | 6.286 | 0.019* |  |
| Percentage of severity 2 feverishness | Overall | 5992 | 705 | 11.8 | 5936 | | 329 | 5.5 | -6.2 | | -7.2 | -5.2 | 145.867 | 0.000*** |  |
|  | 1st half | 2996 | 372 | 12.4 | 2968 | | 180 | 6.1 | -6.4 | | -7.8 | -4.9 | 71.622 | 0.000*** |  |
|  | 2nd half | 2996 | 333 | 11.1 | 2968 | | 149 | 5.0 | -6.1 | | -7.5 | -4.7 | 74.550 | 0.000*** |  |
| Percentage of severity 3 feverishness | Overall | 5992 | 79 | 1.3 | 5936 | | 62 | 1.0 | -0.3 | | -0.7 | 0.1 | 1.916 | 0.176 |  |
|  | 1st half | 2996 | 36 | 1.2 | 2968 | | 34 | 1.1 | -0.1 | | -0.6 | 0.5 | 0.040 | 0.904 |  |
|  | 2nd half | 2996 | 43 | 1.4 | 2968 | | 28 | 0.9 | -0.5 | | -1.0 | 0.1 | 3.066 | 0.094 |  |
| Percentage of severity 4 feverishness | Overall | 5992 | 12 | 0.2 | 5936 | | 21 | 0.4 | 0.2 | | 0.0 | 0.3 | 2.547 | 0.119 |  |
|  | 1st half | 2996 | 1 | 0.0 | 2968 | | 14 | 0.5 | 0.4 | | 0.2 | 0.7 | 11.418 | 0.000*** |  |
|  | 2nd half | 2996 | 11 | 0.4 | 2968 | | 7 | 0.2 | -0.1 | | -0.4 | 0.1 | 0.854 | 0.480 |  |
| Percentage of severity 2 fatigue | Overall | 5992 | 1849 | 30.9 | 5936 | | 1492 | 25.1 | -5.7 | | -7.3 | -4.1 | 48.436 | 0.000*** |  |
|  | 1st half | 2996 | 943 | 31.5 | 2968 | | 785 | 26.4 | -5.0 | | -7.3 | -2.7 | 18.305 | 0.000*** |  |
|  | 2nd half | 2996 | 906 | 30.2 | 2968 | | 707 | 23.8 | -6.4 | | -8.7 | -4.2 | 31.141 | 0.000*** |  |
| Percentage of severity 3 fatigue | Overall | 5992 | 983 | 16.4 | 5936 | | 765 | 12.9 | -3.5 | | -4.8 | -2.2 | 29.503 | 0.000*** |  |
|  | 1st half | 2996 | 484 | 16.2 | 2968 | | 396 | 13.3 | -2.8 | | -4.6 | -1.0 | 9.377 | 0.002** |  |
|  | 2nd half | 2996 | 499 | 16.7 | 2968 | | 369 | 12.4 | -4.2 | | -6.0 | -2.4 | 21.381 | 0.000*** |  |
| Percentage of severity 4 fatigue | Overall | 5992 | 305 | 5.1 | 5936 | | 231 | 3.9 | -1.2 | | -1.9 | -0.5 | 9.982 | 0.002** |  |
|  | 1st half | 2996 | 135 | 4.5 | 2968 | | 139 | 4.7 | 0.2 | | -0.9 | 1.2 | 0.107 | 0.757 |  |
|  | 2nd half | 2996 | 170 | 5.7 | 2968 | | 92 | 3.1 | -2.6 | | -3.6 | -1.5 | 23.529 | 0.000*** |  |
| Percentage of severity 2 sneezing | Overall | 5992 | 1057 | 17.6 | 5936 | | 849 | 14.3 | -3.3 | | -4.7 | -2.0 | 24.742 | 0.000*** |  |
|  | 1st half | 2996 | 529 | 17.7 | 2968 | | 436 | 14.7 | -3.0 | | -4.8 | -1.1 | 9.677 | 0.002** |  |
|  | 2nd half | 2996 | 528 | 17.6 | 2968 | | 413 | 13.9 | -3.7 | | -5.6 | -1.9 | 15.430 | 0.000*** |  |
| Percentage of severity 3 sneezing | Overall | 5992 | 367 | 6.1 | 5936 | | 311 | 5.2 | -0.9 | | -1.7 | -0.1 | 4.363 | 0.040* |  |
|  | 1st half | 2996 | 164 | 5.5 | 2968 | | 127 | 4.3 | -1.2 | | -2.3 | -0.1 | 4.587 | 0.035* |  |
|  | 2nd half | 2996 | 203 | 6.8 | 2968 | | 184 | 6.2 | -0.6 | | -1.8 | 0.7 | 0.816 | 0.372 |  |
| Percentage of severity 4 sneezing | Overall | 5992 | 65 | 1.1 | 5936 | | 40 | 0.7 | -0.4 | | -0.7 | -0.1 | 5.771 | 0.018* |  |
|  | 1st half | 2996 | 29 | 1.0 | 2968 | | 11 | 0.4 | -0.6 | | -1.0 | -0.2 | 7.986 | 0.006** |  |
|  | 2nd half | 2996 | 36 | 1.2 | 2968 | | 29 | 1.0 | -0.2 | | -0.8 | 0.3 | 0.697 | 0.455 |  |
| Percentage of severity 2 runny nose | Overall | 5992 | 1145 | 19.1 | 5936 | | 922 | 15.5 | -3.6 | | -4.9 | -2.2 | 26.624 | 0.000*** |  |
|  | 1st half | 2996 | 609 | 20.3 | 2968 | | 460 | 15.5 | -4.8 | | -6.8 | -2.9 | 23.628 | 0.000*** |  |
|  | 2nd half | 2996 | 536 | 17.9 | 2968 | | 462 | 15.6 | -2.3 | | -4.2 | -0.4 | 5.782 | 0.017* |  |
| Percentage of severity 3 runny nose | Overall | 5992 | 522 | 8.7 | 5936 | | 416 | 7.0 | -1.7 | | -2.7 | -0.7 | 11.944 | 0.001*** |  |
|  | 1st half | 2996 | 234 | 7.8 | 2968 | | 183 | 6.2 | -1.6 | | -2.9 | -0.4 | 6.201 | 0.013* |  |
|  | 2nd half | 2996 | 288 | 9.6 | 2968 | | 233 | 7.9 | -1.8 | | -3.2 | -0.3 | 5.809 | 0.017* |  |
| Percentage of severity 4 runny nose | Overall | 5992 | 149 | 2.5 | 5936 | | 67 | 1.1 | -1.4 | | -1.8 | -0.9 | 30.925 | 0.000*** |  |
|  | 1st half | 2996 | 65 | 2.2 | 2968 | | 45 | 1.5 | -0.7 | | -1.3 | 0.0 | 3.516 | 0.067 |  |
|  | 2nd half | 2996 | 84 | 2.8 | 2968 | | 22 | 0.7 | -2.1 | | -2.7 | -1.4 | 36.331 | 0.000*** |  |
| Percentage of severity 2 nasal congestion | Overall | 5992 | 862 | 14.4 | 5936 | | 574 | 9.7 | -4.7 | | -5.9 | -3.5 | 62.629 | 0.000*** |  |
|  | 1st half | 2996 | 427 | 14.3 | 2968 | | 314 | 10.6 | -3.7 | | -5.3 | -2.0 | 18.484 | 0.000*** |  |
|  | 2nd half | 2996 | 435 | 14.5 | 2968 | | 260 | 8.8 | -5.8 | | -7.4 | -4.1 | 48.036 | 0.000*** |  |
| Percentage of severity 3 nasal congestion | Overall | 5992 | 235 | 3.9 | 5936 | | 150 | 2.5 | -1.4 | | -2.0 | -0.8 | 18.577 | 0.000*** |  |
|  | 1st half | 2996 | 94 | 3.1 | 2968 | | 81 | 2.7 | -0.4 | | -1.3 | 0.4 | 0.873 | 0.358 |  |
|  | 2nd half | 2996 | 141 | 4.7 | 2968 | | 69 | 2.3 | -2.4 | | -3.3 | -1.4 | 24.891 | 0.000*** |  |
| Percentage of severity 4 nasal congestion | Overall | 5992 | 67 | 1.1 | 5936 | | 46 | 0.8 | -0.3 | | -0.7 | 0.0 | 3.744 | 0.059 |  |
|  | 1st half | 2996 | 22 | 0.7 | 2968 | | 21 | 0.7 | 0.0 | | -0.5 | 0.4 | 0.015 | 1.000 |  |
|  | 2nd half | 2996 | 45 | 1.5 | 2968 | | 25 | 0.8 | -0.7 | | -1.2 | -0.1 | 5.594 | 0.022* |  |
| Percentage of severity 2 sore throat | Overall | 5992 | 850 | 14.2 | 5936 | | 372 | 6.3 | -7.9 | | -9.0 | -6.8 | 203.351 | 0.000*** |  |
|  | 1st half | 2996 | 427 | 14.3 | 2968 | | 213 | 7.2 | -7.1 | | -8.6 | -5.5 | 77.925 | 0.000*** |  |
|  | 2nd half | 2996 | 423 | 14.1 | 2968 | | 159 | 5.4 | -8.8 | | -10.3 | -7.3 | 129.973 | 0.000*** |  |
| Percentage of severity 3 sore throat | Overall | 5992 | 180 | 3.0 | 5936 | | 154 | 2.6 | -0.4 | | -1.0 | 0.2 | 1.839 | 0.183 |  |
|  | 1st half | 2996 | 96 | 3.2 | 2968 | | 97 | 3.3 | 0.1 | | -0.8 | 1.0 | 0.019 | 0.942 |  |
|  | 2nd half | 2996 | 84 | 2.8 | 2968 | | 57 | 1.9 | -0.9 | | -1.7 | -0.1 | 5.039 | 0.027* |  |
| Percentage of severity 4 sore throat | Overall | 5992 | 58 | 1.0 | 5936 | | 32 | 0.5 | -0.4 | | -0.7 | -0.1 | 7.324 | 0.008** |  |
|  | 1st half | 2996 | 28 | 0.9 | 2968 | | 24 | 0.8 | -0.1 | | -0.6 | 0.3 | 0.274 | 0.677 |  |
|  | 2nd half | 2996 | 30 | 1.0 | 2968 | | 8 | 0.3 | -0.7 | | -1.1 | -0.3 | 12.612 | 0.000*** |  |
| Percentage of severity 2 coughing | Overall | 5992 | 767 | 12.8 | 5936 | | 510 | 8.6 | -4.2 | | -5.3 | -3.1 | 55.253 | 0.000*** |  |
|  | 1st half | 2996 | 389 | 13.0 | 2968 | | 273 | 9.2 | -3.8 | | -5.4 | -2.2 | 21.656 | 0.000*** |  |
|  | 2nd half | 2996 | 378 | 12.6 | 2968 | | 237 | 8.0 | -4.6 | | -6.2 | -3.1 | 34.583 | 0.000*** |  |
| Percentage of severity 3 coughing | Overall | 5992 | 187 | 3.1 | 5936 | | 119 | 2.0 | -1.1 | | -1.7 | -0.5 | 14.861 | 0.000*** |  |
|  | 1st half | 2996 | 96 | 3.2 | 2968 | | 54 | 1.8 | -1.4 | | -2.2 | -0.6 | 11.663 | 0.001*** |  |
|  | 2nd half | 2996 | 91 | 3.0 | 2968 | | 65 | 2.2 | -0.8 | | -1.7 | 0.0 | 4.203 | 0.043* |  |
| Percentage of severity 4 coughing | Overall | 5992 | 77 | 1.3 | 5936 | | 22 | 0.4 | -0.9 | | -1.2 | -0.6 | 30.293 | 0.000*** |  |
|  | 1st half | 2996 | 33 | 1.1 | 2968 | | 17 | 0.6 | -0.5 | | -1.0 | -0.1 | 5.013 | 0.032* |  |
|  | 2nd half | 2996 | 44 | 1.5 | 2968 | | 5 | 0.2 | -1.3 | | -1.8 | -0.8 | 30.930 | 0.000*** |  |
| Percentage of severity 2 arthralgia | Overall | 5992 | 971 | 16.2 | 5936 | | 517 | 8.7 | -7.5 | | -8.7 | -6.3 | 153.432 | 0.000*** |  |
|  | 1st half | 2996 | 442 | 14.8 | 2968 | | 285 | 9.6 | -5.2 | | -6.8 | -3.5 | 36.952 | 0.000*** |  |
|  | 2nd half | 2996 | 529 | 17.7 | 2968 | | 232 | 7.8 | -9.8 | | -11.5 | -8.1 | 129.691 | 0.000*** |  |
| Percentage of severity 3 arthralgia | Overall | 5992 | 178 | 3.0 | 5936 | | 122 | 2.1 | -0.9 | | -1.5 | -0.4 | 10.191 | 0.002** |  |
|  | 1st half | 2996 | 97 | 3.2 | 2968 | | 63 | 2.1 | -1.1 | | -1.9 | -0.3 | 7.100 | 0.008** |  |
|  | 2nd half | 2996 | 81 | 2.7 | 2968 | | 59 | 2.0 | -0.7 | | -1.5 | 0.1 | 3.332 | 0.073 |  |
| Percentage of severity 4 arthralgia | Overall | 5992 | 23 | 0.4 | 5936 | | 82 | 1.4 | 1.0 | | 0.7 | 1.3 | 34.009 | 0.000*** |  |
|  | 1st half | 2996 | 9 | 0.3 | 2968 | | 47 | 1.6 | 1.3 | | 0.8 | 1.8 | 26.392 | 0.000*** |  |
|  | 2nd half | 2996 | 14 | 0.5 | 2968 | | 35 | 1.2 | 0.7 | | 0.3 | 1.2 | 9.275 | 0.002** |  |
| Percentage of severity 2 myalgia | Overall | 5992 | 948 | 15.8 | 5936 | | 570 | 9.6 | -6.2 | | -7.4 | -5.0 | 103.826 | 0.000*** |  |
|  | 1st half | 2996 | 479 | 16.0 | 2968 | | 312 | 10.5 | -5.5 | | -7.2 | -3.8 | 38.862 | 0.000*** |  |
|  | 2nd half | 2996 | 469 | 15.7 | 2968 | | 258 | 8.7 | -7.0 | | -8.6 | -5.3 | 67.504 | 0.000*** |  |
| Percentage of severity 3 myalgia | Overall | 5992 | 227 | 3.8 | 5936 | | 146 | 2.5 | -1.3 | | -2.0 | -0.7 | 17.381 | 0.000*** |  |
|  | 1st half | 2996 | 110 | 3.7 | 2968 | | 87 | 2.9 | -0.7 | | -1.6 | 0.2 | 2.558 | 0.112 |  |
|  | 2nd half | 2996 | 117 | 3.9 | 2968 | | 59 | 2.0 | -1.9 | | -2.8 | -1.1 | 19.138 | 0.000*** |  |
| Percentage of severity 4 myalgia | Overall | 5992 | 65 | 1.1 | 5936 | | 37 | 0.6 | -0.5 | | -0.8 | -0.1 | 7.490 | 0.007** |  |
|  | 1st half | 2996 | 23 | 0.8 | 2968 | | 22 | 0.7 | 0.0 | | -0.5 | 0.4 | 0.014 | 1.000 |  |
|  | 2nd half | 2996 | 42 | 1.4 | 2968 | | 15 | 0.5 | -0.9 | | -1.4 | -0.4 | 12.658 | 0.000*** |  |
| Percentage of severity 2 headache | Overall | 5992 | 940 | 15.7 | 5936 | | 584 | 9.8 | -5.8 | | -7.0 | -4.7 | 91.550 | 0.000*** |  |
|  | 1st half | 2996 | 460 | 15.4 | 2968 | | 333 | 11.2 | -4.1 | | -5.9 | -2.4 | 22.104 | 0.000*** |  |
|  | 2nd half | 2996 | 480 | 16.0 | 2968 | | 251 | 8.5 | -7.6 | | -9.2 | -5.9 | 79.329 | 0.000*** |  |
| Percentage of severity 3 headache | Overall | 5992 | 266 | 4.4 | 5936 | | 206 | 3.5 | -1.0 | | -1.7 | -0.3 | 7.366 | 0.007** |  |
|  | 1st half | 2996 | 132 | 4.4 | 2968 | | 113 | 3.8 | -0.6 | | -1.6 | 0.4 | 1.356 | 0.267 |  |
|  | 2nd half | 2996 | 134 | 4.5 | 2968 | | 93 | 3.1 | -1.3 | | -2.3 | -0.4 | 7.303 | 0.008** |  |
| Percentage of severity 4 headache | Overall | 5992 | 46 | 0.8 | 5936 | | 61 | 1.0 | 0.3 | | -0.1 | 0.6 | 2.266 | 0.145 |  |
|  | 1st half | 2996 | 18 | 0.6 | 2968 | | 30 | 1.0 | 0.4 | | 0.0 | 0.9 | 3.139 | 0.083 |  |
|  | 2nd half | 2996 | 28 | 0.9 | 2968 | | 31 | 1.0 | 0.1 | | -0.4 | 0.6 | 0.184 | 0.696 |  |

⊿, Difference in the proportion of applicable days (*Euglena* group versus placebo group); 95% CI−, Lower bound of 95% confidence interval; 95% CI+, Upper bound of 95% confidence interval; Between-group comparisons were conducted using the chi-square test, **P* < 0.05, ***P* < 0.01, ****P* < 0.001.

(1) Normal; (2) Slight ; (3) Mild; (4) Moderate; (5) Severe.

**Table S8.** Well-being factors measured with VAS.

| **Item** | **Unit** | **Time** | | **Placebo group** | | | | | |  | ***Euglena* group** | | | | | |  | **Between-group comparisons** | | | | |
| --- | --- | --- | --- | --- | --- | --- | --- | --- | --- | --- | --- | --- | --- | --- | --- | --- | --- | --- | --- | --- | --- | --- |
|  |  |  |  | n | Mean | SD | Med | Min | Max |  | n | Mean | SD | Med | Min | Max |  | ⊿ | SE | 95% CI− | 95% CI+ | *P-*value |
| Physical fatigue | mm | Baseline | * | 107 | 38.4 | 22.2 | 34.0 | 7.0 | 95.0 |  | 106 | 38.7 | 21.9 | 34.0 | 8.0 | 92.0 |  | 0.3 | 3.0 | -5.7 | 6.2 | 0.929 |
|  |  | Week 8 | # | 107 | 34.8 | 21.7 | 30.0 | 0.0 | 80.0 |  | 106 | 34.7 | 23.0 | 32.0 | 0.0 | 82.0 |  | -0.2 | 2.8 | -5.8 | 5.4 | 0.952 |
| Mental fatigue | mm | Baseline | * | 107 | 35.5 | 24.3 | 29.0 | 0.0 | 99.0 |  | 106 | 37.4 | 23.1 | 34.0 | 0.0 | 90.0 |  | 1.9 | 3.3 | -4.5 | 8.3 | 0.559 |
|  |  | Week 8 | # | 107 | 35.0 | 24.1 | 31.0 | 0.0 | 96.0 |  | 106 | 35.0 | 23.9 | 31.5 | 0.0 | 82.0 |  | -0.9 | 3.0 | -6.7 | 5.0 | 0.775 |
| Stress | mm | Baseline | * | 107 | 37.9 | 23.9 | 33.0 | 0.0 | 99.0 |  | 106 | 40.7 | 23.0 | 42.5 | 4.0 | 90.0 |  | 2.8 | 3.2 | -3.5 | 9.2 | 0.376 |
|  |  | Week 8 | # | 107 | 36.1 | 24.3 | 35.0 | 0.0 | 97.0 |  | 106 | 38.5 | 25.2 | 38.5 | 0.0 | 82.0 |  | 0.9 | 2.9 | -4.8 | 6.7 | 0.752 |
| Mood | mm | Baseline | * | 107 | 31.6 | 22.1 | 25.0 | 2.0 | 81.0 |  | 106 | 31.9 | 20.1 | 28.0 | 2.0 | 90.0 |  | 0.3 | 2.9 | -5.4 | 6.0 | 0.921 |
|  |  | Week 8 | # | 107 | 28.4 | 20.5 | 24.0 | 0.0 | 82.0 |  | 106 | 29.3 | 18.9 | 28.0 | 0.0 | 76.0 |  | 0.8 | 2.4 | -4.0 | 5.6 | 0.749 |
| Energy | mm | Baseline | * | 107 | 33.5 | 20.5 | 29.0 | 2.0 | 86.0 |  | 106 | 35.0 | 20.1 | 31.5 | 2.0 | 91.0 |  | 1.5 | 2.8 | -4.0 | 7.0 | 0.591 |
|  |  | Week 8 | # | 107 | 30.7 | 20.8 | 26.0 | 0.0 | 78.0 |  | 106 | 33.0 | 20.9 | 31.0 | 1.0 | 86.0 |  | 1.6 | 2.6 | -3.5 | 6.8 | 0.529 |
| Anxiety | mm | Baseline | * | 107 | 35.6 | 23.2 | 33.0 | 0.0 | 86.0 |  | 106 | 37.1 | 23.7 | 35.5 | 0.0 | 90.0 |  | 1.5 | 3.2 | -4.8 | 7.9 | 0.634 |
|  |  | Week 8 | # | 107 | 32.3 | 22.4 | 33.0 | 0.0 | 88.0 |  | 106 | 32.8 | 23.2 | 29.0 | 0.0 | 89.0 |  | -0.1 | 2.9 | -5.8 | 5.5 | 0.971 |
| Tension | mm | Baseline | * | 107 | 29.3 | 22.2 | 21.0 | 0.0 | 86.0 |  | 106 | 33.3 | 22.1 | 31.5 | 0.0 | 90.0 |  | 4.0 | 3.0 | -1.9 | 10.0 | 0.183 |
|  |  | Week 8 | # | 107 | 25.4 | 18.5 | 21.0 | 0.0 | 73.0 |  | 106 | 26.9 | 20.2 | 23.5 | 0.0 | 89.0 |  | 0.2 | 2.5 | -4.7 | 5.0 | 0.945 |
| Depression | mm | Baseline | * | 107 | 30.0 | 24.3 | 21.0 | 0.0 | 88.0 |  | 106 | 31.1 | 22.6 | 23.5 | 0.0 | 91.0 |  | 1.1 | 3.2 | -5.2 | 7.5 | 0.730 |
|  |  | Week 8 | # | 107 | 26.6 | 21.1 | 21.0 | 0.0 | 91.0 |  | 106 | 26.6 | 22.2 | 19.0 | 0.0 | 98.0 |  | -0.3 | 2.8 | -5.9 | 5.3 | 0.914 |
| Relaxedness | mm | Baseline | * | 107 | 28.0 | 21.9 | 23.0 | 0.0 | 92.0 |  | 106 | 33.2 | 21.3 | 31.0 | 0.0 | 90.0 |  | 5.2 | 3.0 | -0.6 | 11.0 | 0.081 |
|  |  | Week 8 | # | 107 | 25.6 | 19.1 | 20.0 | 0.0 | 78.0 |  | 106 | 28.0 | 20.4 | 24.5 | 0.0 | 83.0 |  | 0.5 | 2.5 | -4.4 | 5.5 | 0.837 |
| Irritability | mm | Baseline | * | 107 | 31.7 | 23.8 | 25.0 | 0.0 | 92.0 |  | 106 | 30.7 | 23.8 | 24.0 | 0.0 | 94.0 |  | -0.9 | 3.3 | -7.3 | 5.5 | 0.781 |
|  |  | Week 8 | # | 107 | 27.4 | 22.7 | 20.0 | 0.0 | 83.0 |  | 106 | 26.3 | 21.5 | 21.5 | 0.0 | 89.0 |  | -0.8 | 2.8 | -6.3 | 4.7 | 0.786 |
| Satisfaction with sleep | mm | Baseline | * | 107 | 40.9 | 24.1 | 43.0 | 0.0 | 97.0 |  | 106 | 42.6 | 23.6 | 46.5 | 3.0 | 94.0 |  | 1.7 | 3.3 | -4.8 | 8.1 | 0.606 |
|  |  | Week 8 | # | 107 | 36.8 | 25.6 | 34.0 | 0.0 | 99.0 |  | 106 | 39.4 | 25.1 | 35.5 | 0.0 | 98.0 |  | 1.7 | 3.1 | -4.3 | 7.7 | 0.579 |
| Wake-up quality | mm | Baseline | * | 107 | 36.6 | 24.0 | 34.0 | 0.0 | 98.0 |  | 106 | 39.3 | 22.4 | 40.0 | 0.0 | 94.0 |  | 2.8 | 3.2 | -3.5 | 9.0 | 0.387 |
|  |  | Week 8 | # | 107 | 31.2 | 23.1 | 24.0 | 0.0 | 89.0 |  | 106 | 35.9 | 25.5 | 30.0 | 0.0 | 88.0 |  | 3.6 | 3.1 | -2.5 | 9.7 | 0.241 |
| Falling asleep | mm | Baseline | * | 107 | 31.0 | 24.4 | 25.0 | 0.0 | 92.0 |  | 106 | 34.5 | 24.0 | 31.5 | 0.0 | 94.0 |  | 3.5 | 3.3 | -3.0 | 10.1 | 0.292 |
|  |  | Week 8 | # | 107 | 27.6 | 25.3 | 18.0 | 0.0 | 99.0 |  | 106 | 31.1 | 23.6 | 27.0 | 0.0 | 86.0 |  | 1.8 | 3.0 | -4.1 | 7.7 | 0.540 |
| Satisfaction with defecation | mm | Baseline | * | 107 | 35.2 | 24.4 | 36.0 | 0.0 | 88.0 |  | 106 | 39.1 | 26.6 | 33.0 | 0.0 | 100.0 |  | 3.9 | 3.5 | -2.9 | 10.8 | 0.260 |
|  |  | Week 8 | # | 107 | 30.4 | 23.5 | 28.0 | 0.0 | 95.0 |  | 106 | 30.8 | 25.5 | 23.0 | 0.0 | 93.0 |  | -1.7 | 2.9 | -7.3 | 4.0 | 0.561 |
| Refreshment upon defecation | mm | Baseline | * | 107 | 31.9 | 23.4 | 27.0 | 0.0 | 85.0 |  | 106 | 37.9 | 25.2 | 35.0 | 0.0 | 100.0 |  | 6.0 | 3.3 | -0.6 | 12.5 | 0.076 |
|  |  | Week 8 | # | 107 | 28.9 | 22.3 | 21.0 | 0.0 | 94.0 |  | 106 | 29.8 | 24.9 | 23.5 | 0.0 | 97.0 |  | -2.6 | 2.6 | -7.7 | 2.5 | 0.322 |

Max, Maximum; Med, Median; Min, Minimum; SD, Standard deviation; SE, Standard error of the difference between groups. n, Number of participants; ⊿, Difference between groups (*Euglena* group versus placebo group); 95% CI−, Lower bound of 95% confidence interval; 95% CI+, Upper bound of 95% confidence interval; *Between-group comparisons with Welch’s *t*-test; #Between-group comparisons with ANCOVA with baseline as a covariate and group as a factor (Δ is defined as between-group differences in estimated marginal means).

**Table S9.** Well-being factors measured with VAS (comparisons within the placebo group).

| **Item** | **Unit** | **Baseline** | | | | | |  | **Week 8** | | | | | |  | **Within-group comparisons *** | | | | |
| --- | --- | --- | --- | --- | --- | --- | --- | --- | --- | --- | --- | --- | --- | --- | --- | --- | --- | --- | --- | --- |
|  |  | n | Mean | SD | Med | Min | Max |  | n | Mean | SD | Med | Min | Max |  | ⊿ | SE | 95% CI− | 95% CI+ | *P*-value |
| Physical fatigue | mm | 107 | 38.4 | 22.2 | 34.0 | 7.0 | 95.0 |  | 107 | 34.8 | 21.7 | 30.0 | 0.0 | 80.0 |  | -3.6 | 2.2 | -8.0 | 0.8 | 0.108 |
| Mental fatigue | mm | 107 | 35.5 | 24.3 | 29.0 | 0.0 | 99.0 |  | 107 | 35.0 | 24.1 | 31.0 | 0.0 | 96.0 |  | -0.4 | 2.3 | -4.9 | 4.0 | 0.852 |
| Stress | mm | 107 | 37.9 | 23.9 | 33.0 | 0.0 | 99.0 |  | 107 | 36.1 | 24.3 | 35.0 | 0.0 | 97.0 |  | -1.8 | 2.2 | -6.1 | 2.5 | 0.405 |
| Mood | mm | 107 | 31.6 | 22.1 | 25.0 | 2.0 | 81.0 |  | 107 | 28.4 | 20.5 | 24.0 | 0.0 | 82.0 |  | -3.2 | 2.1 | -7.3 | 1.0 | 0.136 |
| Energy | mm | 107 | 33.5 | 20.5 | 29.0 | 2.0 | 86.0 |  | 107 | 30.7 | 20.8 | 26.0 | 0.0 | 78.0 |  | -2.8 | 2.0 | -6.7 | 1.1 | 0.153 |
| Anxiety | mm | 107 | 35.6 | 23.2 | 33.0 | 0.0 | 86.0 |  | 107 | 32.3 | 22.4 | 33.0 | 0.0 | 88.0 |  | -3.2 | 2.2 | -7.5 | 1.1 | 0.141 |
| Tension | mm | 107 | 29.3 | 22.2 | 21.0 | 0.0 | 86.0 |  | 107 | 25.4 | 18.5 | 21.0 | 0.0 | 73.0 |  | -3.9 | 2.1 | -8.1 | 0.3 | 0.066 |
| Depression | mm | 107 | 30.0 | 24.3 | 21.0 | 0.0 | 88.0 |  | 107 | 26.6 | 21.1 | 21.0 | 0.0 | 91.0 |  | -3.4 | 2.5 | -8.3 | 1.5 | 0.172 |
| Relaxedness | mm | 107 | 28.0 | 21.9 | 23.0 | 0.0 | 92.0 |  | 107 | 25.6 | 19.1 | 20.0 | 0.0 | 78.0 |  | -2.4 | 2.1 | -6.6 | 1.7 | 0.247 |
| Irritability | mm | 107 | 31.7 | 23.8 | 25.0 | 0.0 | 92.0 |  | 107 | 27.4 | 22.7 | 20.0 | 0.0 | 83.0 |  | -4.3 | 2.4 | -9.1 | 0.5 | 0.078 |
| Satisfaction with sleep | mm | 107 | 40.9 | 24.1 | 43.0 | 0.0 | 97.0 |  | 107 | 36.8 | 25.6 | 34.0 | 0.0 | 99.0 |  | -4.0 | 2.2 | -8.5 | 0.4 | 0.072 |
| Wake-up quality | mm | 107 | 36.6 | 24.0 | 34.0 | 0.0 | 98.0 |  | 107 | 31.2 | 23.1 | 24.0 | 0.0 | 89.0 |  | -5.4 | 2.5 | -10.3 | -0.5 | 0.031* |
| Falling asleep | mm | 107 | 31.0 | 24.4 | 25.0 | 0.0 | 92.0 |  | 107 | 27.6 | 25.3 | 18.0 | 0.0 | 99.0 |  | -3.4 | 2.3 | -7.9 | 1.1 | 0.142 |
| Satisfaction with defecation | mm | 107 | 35.2 | 24.4 | 36.0 | 0.0 | 88.0 |  | 107 | 30.4 | 23.5 | 28.0 | 0.0 | 95.0 |  | -4.8 | 2.4 | -9.6 | 0.0 | 0.049* |
| Refreshment upon defecation | mm | 107 | 31.9 | 23.4 | 27.0 | 0.0 | 85.0 |  | 107 | 28.9 | 22.3 | 21.0 | 0.0 | 94.0 |  | -3.0 | 2.1 | -7.3 | 1.2 | 0.160 |

Max, Maximum; Med, Median; Min, Minimum; SD, Standard deviation; SE, Standard error of within-group differences. n: Number of participants; ⊿, Within-group difference (Week 8 versus Baseline); 95% CI−, Lower bound of 95% confidence interval; 95% CI+, Upper bound of 95% confidence interval; Within-group comparisons were conducted with paired *t*-test, **P <* 0.05.

**Table S10.** Well-being factors measured with VAS (comparisons within the *Euglena* group).

| **Item** | **Unit** | **Baseline** | | | | | |  | **Week 8** | | | | | |  | **Within-group comparisons** | | | | |
| --- | --- | --- | --- | --- | --- | --- | --- | --- | --- | --- | --- | --- | --- | --- | --- | --- | --- | --- | --- | --- |
|  |  | n | Mean | SD | Med | Min | Max |  | n | Mean | SD | Med | Min | Max |  | Δ | SE | 95% CI− | 95% CI+ | *P*-value |
| Physical fatigue | mm | 106 | 38.7 | 21.9 | 34.0 | 8.0 | 92.0 |  | 106 | 34.7 | 23.0 | 32.0 | 0.0 | 82.0 |  | -3.9 | 2.5 | -9.0 | 1.1 | 0.124 |
| Mental fatigue | mm | 106 | 37.4 | 23.1 | 34.0 | 0.0 | 90.0 |  | 106 | 35.0 | 23.9 | 31.5 | 0.0 | 82.0 |  | -2.3 | 2.6 | -7.6 | 2.9 | 0.380 |
| Stress | mm | 106 | 40.7 | 23.0 | 42.5 | 4.0 | 90.0 |  | 106 | 38.5 | 25.2 | 38.5 | 0.0 | 82.0 |  | -2.2 | 2.4 | -7.0 | 2.6 | 0.366 |
| Mood | mm | 106 | 31.9 | 20.1 | 28.0 | 2.0 | 90.0 |  | 106 | 29.3 | 18.9 | 28.0 | 0.0 | 76.0 |  | -2.5 | 2.1 | -6.8 | 1.7 | 0.233 |
| Energy | mm | 106 | 35.0 | 20.1 | 31.5 | 2.0 | 91.0 |  | 106 | 33.0 | 20.9 | 31.0 | 1.0 | 86.0 |  | -2.0 | 2.3 | -6.7 | 2.6 | 0.386 |
| Anxiety | mm | 106 | 37.1 | 23.7 | 35.5 | 0.0 | 90.0 |  | 106 | 32.8 | 23.2 | 29.0 | 0.0 | 89.0 |  | -4.2 | 2.7 | -9.6 | 1.1 | 0.121 |
| Tension | mm | 106 | 33.3 | 22.1 | 31.5 | 0.0 | 90.0 |  | 106 | 26.9 | 20.2 | 23.5 | 0.0 | 89.0 |  | -6.4 | 2.4 | -11.1 | -1.7 | 0.008** |
| Depression | mm | 106 | 31.1 | 22.6 | 23.5 | 0.0 | 91.0 |  | 106 | 26.6 | 22.2 | 19.0 | 0.0 | 98.0 |  | -4.5 | 2.6 | -9.7 | 0.7 | 0.092 |
| Relaxedness | mm | 106 | 33.2 | 21.3 | 31.0 | 0.0 | 90.0 |  | 106 | 28.0 | 20.4 | 24.5 | 0.0 | 83.0 |  | -5.2 | 2.3 | -9.8 | -0.6 | 0.026* |
| Irritability | mm | 106 | 30.7 | 23.8 | 24.0 | 0.0 | 94.0 |  | 106 | 26.3 | 21.5 | 21.5 | 0.0 | 89.0 |  | -4.5 | 2.5 | -9.4 | 0.5 | 0.076 |
| Satisfaction with sleep | mm | 106 | 42.6 | 23.6 | 46.5 | 3.0 | 94.0 |  | 106 | 39.4 | 25.1 | 35.5 | 0.0 | 98.0 |  | -3.2 | 2.6 | -8.4 | 2.1 | 0.231 |
| Wake-up quality | mm | 106 | 39.3 | 22.4 | 40.0 | 0.0 | 94.0 |  | 106 | 35.9 | 25.5 | 30.0 | 0.0 | 88.0 |  | -3.4 | 2.6 | -8.6 | 1.8 | 0.196 |
| Falling asleep | mm | 106 | 34.5 | 24.0 | 31.5 | 0.0 | 94.0 |  | 106 | 31.1 | 23.6 | 27.0 | 0.0 | 86.0 |  | -3.4 | 2.6 | -8.6 | 1.8 | 0.197 |
| Satisfaction with defecation | mm | 106 | 39.1 | 26.6 | 33.0 | 0.0 | 100.0 |  | 106 | 30.8 | 25.5 | 23.0 | 0.0 | 93.0 |  | -8.4 | 2.3 | -12.9 | -3.9 | 0.000*** |
| Refreshment upon defecation | mm | 106 | 37.9 | 25.2 | 35.0 | 0.0 | 100.0 |  | 106 | 29.8 | 24.9 | 23.5 | 0.0 | 97.0 |  | -8.0 | 2.0 | -11.9 | -4.2 | 0.000*** |

Max, Maximum; Med, Median; Min, Minimum; SD, Standard deviation; SE, Standard error of within-group differences. n, Number of participants; Δ, Within-group difference (Week 8 versus Baseline); 95% CI−, Lower bound of 95% confidence interval; 95% CI+, Upper bound of 95% confidence interval; Within-group comparisons conducted with paired *t*-test, **P* < 0.05, ***P* < 0.01, ****P* < 0.001.
